# Biochar-Associated Shifts in Nitrogen Status, Microbial Communities and Dissolved Organic Matter in Salt-Affected Maize Soil

**DOI:** 10.3390/microorganisms14091907

**Published:** 2026-08-28

**Authors:** Rui Li, Chi Zhang, Yu Miao, Fangze Li, Ge Zhang, Qiwei Sun, Tianci Hua, Zhikun Pang, Xingjie Lin

**Affiliations:** 1 BGRIMM Technology Group, Institute Environment Engineering, Beijing 100160, China; zhangchiustb@foxmail.com (C.Z.); 491637611@163.com (Y.M.); lfze2016@163.com (F.L.); zhanggling@163.com (G.Z.); b20200050@xs.ustb.edu.cn (Q.S.); htc0101@163.com (T.H.); pzk728@163.com (Z.P.); xingjielin@sina.com (X.L.); 2 Research Center for Eco-Environmental Sciences, Chinese Academy of Sciences, Beijing 100085, China; 3 University of Chinese Academy of Sciences, Beijing 100049, China; 4 School of Energy and Environmental Engineering, University of Science and Technology Beijing, Beijing 100083, China

**Keywords:** biochar, salt-affected soil, maize, rhizosphere, randomized complete block design, qPCR, microbial community, dissolved organic matter, FT-ICR MS

## Abstract

Salt-affected soil constrains maize establishment, and rhizosphere and non-rhizosphere responses to soil biochar remain difficult to establish. We evaluated soil physical and nitrogen properties, qPCR marker genes, 16S and ITS communities, and molecular profiles of dissolved organic matter (DOM) in a 90-day randomized complete block pot experiment (four biochar rates; five blocks; 20 pots). Control maize did not survive; control pots therefore yielded only non-rhizosphere soil, whereas rhizosphere and non-rhizosphere samples from biochar pots were paired. Block-adjusted comparisons showed higher water content and porosity and lower bulk density in all biochar non-rhizosphere groups than in the control (Holm-adjusted *p* < 0.05). Biochar-associated shifts in mineral-N partitioning and selected DNA-level marker-gene abundances accompanied design-aware community differences for NR treatment, R dose and pooled compartment (16S R^2^ = 0.360, 0.435 and 0.218; ITS R^2^ = 0.720, 0.712 and 0.304; all *p* < 0.001); fungal differences also included heterogeneous dispersion. D90 FT-ICR MS profiles described relative DOM molecular variation, but cross-layer residual associations did not survive false-discovery rate correction. Thus, biochar-associated physical and nitrogen changes coincided with microbial and relative DOM restructuring under the tested pot conditions. Because control survival was confounded with treatment, these are observed-group associations rather than pure causal biochar effects.

## 1. Introduction

Salt-affected cropland constrains maize production through osmotic stress, ion imbalance, weak aggregation and disrupted nutrient cycling. These constraints also shape the rhizosphere, where root inputs, water status and microbial selection intersect [1,2,3,4,5,6,7,8]. Amendment responses can therefore differ between root-adhering and non-rhizosphere soil, and both compartments must be interpreted in relation to the plant condition that generated them.

Biochar can alter water retention, pore structure, bulk density, ion buffering and nutrient retention, but its effects depend on feedstock, pyrolysis, soil salinity, dose and crop context [9,10,11,12,13,14,15,16]. Dose-resolved evidence is therefore needed before amendment-associated physical changes can be connected to nutrient and microbial responses.

The measured layers answer complementary questions. Soil physical variables characterize the habitat experienced by roots and microorganisms; mineral and dissolved nitrogen pools describe resource partitioning; and bacterial and fungal communities indicate whether this habitat is accompanied by compositional restructuring [6,7,8,17,18,19,20,21,22,23]. Characterizing these layers together can identify soil-process responses relevant to maize establishment, although it cannot by itself demonstrate improved yield or field performance.

Functional marker genes and DOM molecular traits provide additional bounded context. Copies of *amoA*, *nirK*, *nirS*, *nosZ*, *mcrA* and *pmoA* indicate the abundance of DNA targets carried by nitrogen- and methane-cycling groups, not transcription or process rates [24,25,26]. FT-ICR MS resolves relative molecular fingerprints of the ionizable DOM fraction, while exploratory assembly and co-abundance summaries are sensitive to model assumptions and compositionality [27,28,29,30,31,32,33,34,35,36,37,38,39,40,41,42,43,44,45,46]. Greenhouse-gas responses to biochar are also context dependent [47] and were not included in the numerical synthesis reported here. Across environmental studies, DNA-level functional-gene abundance is commonly only weakly related to the corresponding process rate [48].

We therefore tested whether biochar-associated changes in soil physical conditions and nitrogen status were accompanied by compartment-specific changes in qPCR marker-gene abundance, bacterial and fungal community structure, and relative DOM molecular profiles in one salt-affected maize pot experiment. We predicted that (i) biochar-associated non-rhizosphere groups would differ from the control in water, pore structure and mineral-N partitioning, and (ii) paired rhizosphere and non-rhizosphere samples from biochar-treated pots would show distinct marker-gene and community profiles. Cross-layer associations were treated as hypothesis-generating rather than as tests of a causal mechanism.

## 2. Materials and Methods

### 2.1. Pot Experiment, Experimental Unit and Greenhouse Management

**Figure 1:**
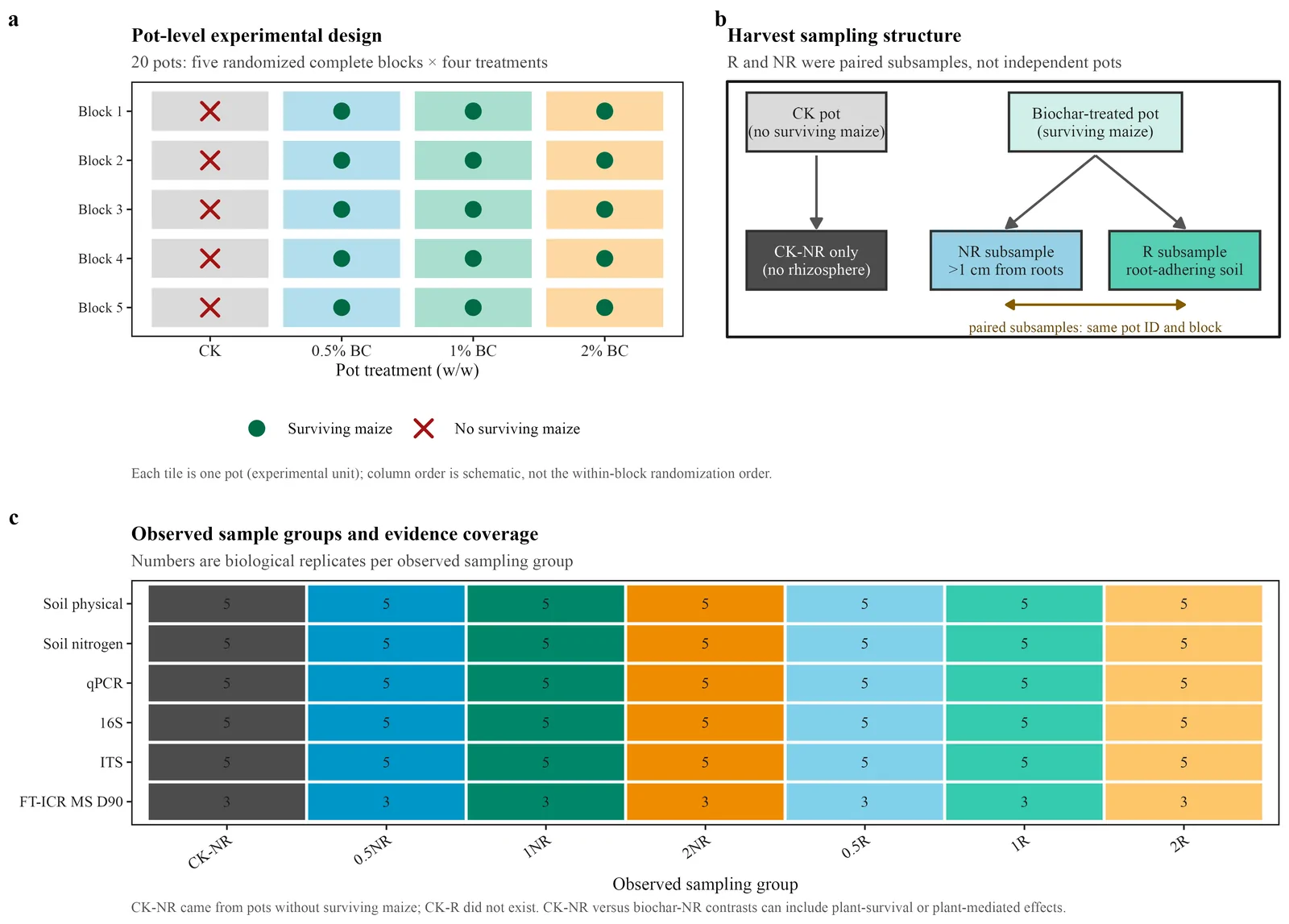
Pot-level design, harvest sampling and evidence coverage. (**a**) The randomized complete block design contained five blocks and four pot treatments (CK, 0.5%, 1% and 2% biochar; 20 pots). Each tile is one experimental unit; treatment columns are schematic and do not represent the recorded within-block randomization order. Green circles indicate surviving maize and red crosses indicate that CK maize did not survive. (**b**) CK pots yielded non-rhizosphere soil only (CK-NR). Each biochar-treated pot yielded paired non-rhizosphere soil (NR; >1 cm from roots) and root-adhering rhizosphere soil (R) with the same pot and block identifier. (**c**) Biological replication for the seven observed sampling groups. Soil physical, soil nitrogen, qPCR, 16S and ITS data had *n* = 5 per group; D90 FT-ICR MS had *n* = 3. CK-NR versus biochar-NR contrasts may contain plant-survival or plant-mediated effects.

The experiment was conducted in a greenhouse in Xiong’an, Hebei, China (38°53′34″ N, 116°1′5″ E), using salt-affected soil collected from the Yellow River Delta (initial pH 8.83; electrical conductivity 2.41 dS m^−1^). Air-dried soil was passed through a 2 mm sieve and 8 kg was placed in each plastic pot (20 cm internal diameter and height; drainage holes and trays). Biochar was mixed at four rates through the full soil mass: CK, 0.5%, 1% and 2% (*w*/*w*), equivalent to 0, 5, 10 and 20 g kg^−1^ and 0, 40, 80 and 160 g pot^−1^, respectively. The randomized complete block design comprised five blocks and one pot per treatment in each block (20 pots; Figure 1); the pot was the treatment experimental unit. Within each block, the four treatments were assigned to pot positions using a random-number table (lot drawing). Pot positions were re-randomized weekly throughout the experiment to reduce persistent light, temperature-gradient and edge-position effects [49].

Three seeds of maize (*Zea mays* L. cv. Zhengdan 958) were sown 3 cm deep on 11 April 2023 and seedlings were thinned to one plant per pot at V3. Pots were maintained at 25 ± 3 °C under natural light. Irrigation water was added gravimetrically to maintain 60–70% water-holding capacity, daily during the warmest period and otherwise every 2–3 days. Tap water was used; no mineral fertilizer, insecticide or fungicide was applied. Harvest and destructive soil sampling occurred on 10 July 2023 (D90), approximately one month after tasseling. Maize did not survive in the CK pots. Consequently, CK supplied non-rhizosphere pot soil only and did not generate a true rhizosphere sample.

### 2.2. Soil Sampling and Design Boundary

At D90, rhizosphere soil (R) was operationally defined as soil remaining attached to roots after gentle shaking; non-rhizosphere soil (NR) was collected more than 1 cm from roots. R and NR were paired subsamples from the same biochar-treated pot and shared its treatment and block identifier. CK-NR samples came from pots without surviving maize. The molecular and soil datasets therefore contained 35 samples in seven observed sampling groups (CK-NR, 0.5NR, 1NR, 2NR, 0.5R, 1R and 2R; *n* = 5 per group) but only 20 independent pots. Approximately 500 g of each sample was sieved to 2 mm and divided for storage at −80 °C (DNA), 4 °C (fresh-soil assays) or after air drying (physicochemical analysis). Because treatment was inseparable from plant survival in CK-NR versus biochar-NR comparisons, these contrasts are termed observed-group or biochar-associated contrasts and may include effects related to plant-survival and plant-mediated processes.

### 2.3. Biochar Characterization

Maize–straw biochar was produced under limited oxygen at 500 °C (10 °C min^−1^ heating rate; 2 h residence time) after the feedstock had been air-dried and ground to <2 mm. The recorded yield was 32.5%. The biochar had pH 9.52, electrical conductivity 3.87 dS m^−1^, total organic carbon 63.14%, total nitrogen 0.34%, C/N 185, ash 16.1%, fixed carbon 61.6%, BET surface area 24.8 m^2^ g^−1^ and cation-exchange capacity 28.5 cmol kg^−1^; the complete characterization is given in Table S1.

### 2.4. Soil Physical Properties and Nitrogen Pools

Soil water content was determined gravimetrically by oven-drying to constant mass. Bulk density and total porosity were obtained by the core method, and redox potential (Eh) was measured using a platinum electrode and expressed relative to the standard hydrogen electrode. Fresh soil was extracted with 2 mol L^−1^ KCl. NH_4_^+^–N was quantified by indophenol-blue colorimetry at 630 nm using a method adapted from HJ 634-2012 [50]; the 2 mol L^−1^ KCl concentration followed the experimental record rather than the 1 mol L^−1^ concentration specified in that standard. NO_3_^−^–N was quantified with a UV–visible spectrophotometer at 220 and 275 nm, with correction for dissolved organic matter absorbance, following GB/T 32737-2016 [51]. TDN in an aqueous extract was determined after alkaline potassium persulfate digestion at 120–124 °C from UV absorbance at 220 and 275 nm, following HJ 636-2012 [52]. DON was calculated by subtraction as TDN − (NH_4_^+^–N + NO_3_^−^–N) [53], and the NH_4_^+^/NO_3_^−^ ratio was calculated from the measured mineral-N concentrations. All N concentrations were expressed on a dry-soil basis.

### 2.5. DNA Extraction and Quality Control

DNA was extracted from 0.5 g fresh soil with the E.Z.N.A. Soil DNA Kit (D5625-02; Omega Bio-tek, Norcross, GA, USA). Soil was bead-beaten for 4 min in SLX-Mlus buffer, treated sequentially with P2 buffer and cHTR reagent to remove humic inhibitors, bound to a HiBind DNA column, washed once with HBC buffer and twice with DNA Wash Buffer, and eluted after addition of 70 µL prewarmed elution buffer. The recovered volume was approximately 65 µL. DNA was assessed using a Qubit 3.0 Fluorometer and NanoDrop spectrophotometry (both Thermo Fisher Scientific, Waltham, MA, USA) and 1.2% agarose gel electrophoresis. Two extraction batches (18 and 17 samples) each included one extraction blank; both blanks contained <0.1 ng µL^−1^ DNA, produced no qPCR amplification and were not sequenced. The same extracts were used for qPCR and amplicon library preparation. The complete extraction steps are provided in Supplementary Method S1.2.

### 2.6. qPCR Marker-Gene Analysis

Reporting followed MIQE principles [54]. The qPCR panel targeted archaeal and bacterial *amoA*, *nirK*, *nirS*, *nosZ*, *mcrA* and *pmoA* using the sequences and sources detailed in Table S11 [55,56,57,58,59,60,61,62,63]. Reactions were run on a CFX96 Real-Time PCR Detection System (Bio-Rad Laboratories, Hercules, CA, USA) with TB Green Premix Ex Taq II (Takara Bio Inc., Kusatsu, Shiga, Japan) in 20 µL: 10 µL premix, each primer at 0.4 µM, 1 µL of 1:10-diluted template (approximately 3–12 ng) and nuclease-free water. Cycling comprised 95 °C for 3 min; 40 cycles of 95 °C for 15 s, gene-specific annealing for 30 s and 72 °C for 30 s; and a 65–95 °C melt curve in 0.5 °C increments every 5 s. Primer sequences, annealing temperatures and amplicon lengths are reported in Table S11.

Tsingke Biotech (Beijing, China) order TS-20231120-0821 and the associated qPCR plate-map records identified FlaCu/R3Cu for *nirK* and cd3aF/R3cd for *nirS*. Table S11 therefore reports the literal sequences and supplier-order names while distinguishing them from published sequence identities: the recorded reverse sequences also match *nirK*1040R-type and *nirS*6QR-type sequences used in other designs, so R3Cu and R3cd are not attributed unqualifiedly to one original published primer pair.

Seven-point plasmid standards (10^1^–10^7^ copies µL^−1^; pMD19-T clones confirmed by sequencing) were run in triplicate on each plate. Standard-curve efficiencies were 95.8–101.2% with R^2^ ≥ 0.994; the limit of detection was 10 copies µL^−1^ and the limit of quantification was 100 copies µL^−1^. Three no-template controls and the two extraction blanks showed no amplification. Three technical replicates were analyzed per sample. If their Cq SD was ≥0.5, the plate was repeated once; if the criterion was still exceeded, the median copy number from the two determinations was retained. A 1:10 versus 1:50 dilution check in three samples per plate gave an approximately 2.3-cycle shift. Copies g^−1^ dry soil were calculated from the 1:10 dilution, the recovered 65 µL extract volume and the dry mass represented by 0.5 g fresh soil after measured-moisture correction, and then log_10_-transformed.

### 2.7. Amplicon Sequencing and Bioinformatics

The V3–V4 region of bacterial 16S rRNA genes was amplified with 338F/806R, and fungal ITS2 with ITS3/ITS4. Two-step PCR added Nextera-compatible dual indices and independent 12 bp sample barcodes. Illumina bcl2fastq2 v2.20.0 first separated the 16S and ITS libraries with zero index mismatches. Cutadapt 4.4 then demultiplexed the anchored 12 bp barcode at the 5′ end of R1 using the exact-match options -g ^{BARCODE}, --no-indels, --error-rate 0 and --discard-untrimmed; unmatched reads were discarded, the barcode was removed and R1 and R2 were oriented to the forward and reverse primer reads, respectively. Libraries were quantified with a Qubit 4.0 Fluorometer (Thermo Fisher Scientific, Waltham, MA, USA), checked with a Qsep400 system (BiOptic Inc., New Taipei City, Taiwan) and sequenced by Magigene (Guangzhou, China) on an Illumina NovaSeq 6000 instrument (Illumina, San Diego, CA, USA) in PE250 mode (35 libraries per marker). Delivered clean-read ranges were 97,098–178,077 for 16S and 98,123–249,880 for ITS; Q30 was 95.2–95.9% and 94.9–95.7%, respectively.

Following barcode removal, reads were processed with fastp 0.23.2 using Q20, a minimum retained length of 150 bp and adapter removal [64]. Paired reads were merged with VSEARCH 2.22.1 using --fastq_mergepairs, --fastq_maxdiffs 15 and --fastq_minovlen 10; ITS reads additionally used --fastq_allowstaggered [65]. Cutadapt 4.4 removed both primer sequences at an allowed error rate of 0.2, and reads without detected primers were discarded [66]. VSEARCH then applied --fastq_maxee 1.0 and minimum lengths of 300 bp for 16S and 200 bp for ITS. After full-length dereplication with size annotations and a minimum unique abundance of 2, UNOISE3 was run in USEARCH v11.0.667 as usearch11 -unoise3 derep.fa -zotus zotus.fa -minsize 8. Because -unoise_alpha was not set, the documented USEARCH default of 2.0 applied [67]. UNOISE3 performed abundance-aware denoising and de novo chimera removal to generate zero-radius operational taxonomic units (zOTUs; exact-sequence features). Representative sequences were additionally screened with VSEARCH --uchime3_denovo, and filtered reads were mapped to zOTU centroids at ≥99% identity with --usearch_global. The delivered feature tables were labeled as ASV tables but contained zOTU identifiers; zOTU is therefore used here. VSEARCH SINTAX 2.22.1 assigned taxonomy at a confidence cutoff of 0.8 [68] against silva_138.1_ssuref_nr99_sintax.fa for 16S [69] and unite_2022-11-28_sh_refs_dynamic_sintax.fa for ITS [70]. Chloroplast and mitochondrial 16S features were removed, and ITS analyses retained features assigned within Fungi. Representative sequences were aligned with MAFFT 7.505 (--auto) [71], trimmed with trimAl 1.4.rev22 (-automated1) [72] and used to construct separate 16S and ITS trees with FastTree 2.1.11 under nucleotide GTR settings [73,74]. Demultiplexing and downstream processing followed the workflow recorded by the sequencing provider, and the local vendor QC reports confirm barcode-oriented preprocessing and delivered read-quality summaries. The date embedded in the ITS filename is therefore not interpreted as an official UNITE release date.

Alpha-diversity tables were generated after single rarefaction without replacement to 60,000 reads per sample for 16S and 50,000 for ITS using vegan::rrarefy with seed 20260620. Rarefaction curves used 1000, 5000, 10,000, 20,000, 30,000, 40,000, 50,000, 60,000 and 70,000 reads, with 100 resamplings per depth. Observed richness, Chao1, Shannon, Simpson and Pielou evenness were calculated with vegan 2.7-5; Faith’s phylogenetic diversity was calculated with picante 1.8.2 and the marker-specific FastTree trees, while Good’s coverage was retained from the delivered alpha-diversity table. For the primary beta-diversity analysis, rarefied counts were converted to relative abundance before Bray–Curtis distances were calculated; analyses using unrarefied relative abundance gave the same significance directions. Relative-abundance summaries were descriptive, and centered log-ratio profiles were retained as a compositional sensitivity view. Kruskal–Wallis tests with false-discovery rate adjustment were used only to screen candidate genera; no candidate was treated as confirmed differential abundance.

### 2.8. FT-ICR MS Analysis of Relative DOM Molecular Profiles

Water-extractable DOM was isolated by solid-phase extraction and analyzed with a Bruker SolariX 12-T Fourier-transform ion cyclotron resonance mass spectrometer (Bruker Daltonics GmbH & Co. KG, Bremen, Germany) using negative-mode electrospray ionization over *m*/*z* 150–1200. Monoisotopic [M−H]^−^ peaks were assigned within <1.0 ppm using C_1–60_H_1–120_O_1–30_N_0–5_S_0–3_, screened by 0.3 ≤ H/C ≤ 2.5, 0 ≤ O/C ≤ 1.2, integer double-bond-equivalent and valence rules, a final S/N ≥ 6 and CH_2_ homologous series of at least three members (Kendrick mass-defect tolerance ±0.001). After blank subtraction, each peak list was standardized to 200 assigned formulas. The manuscript uses D90 samples only (*n* = 3 per observed group). Formula richness, O/C, H/C, DBE, DBE−O, modified aromaticity index and nominal oxidation state of carbon (NOSC) were calculated from assigned formulas. Intensities were normalized within sample and describe the detected ionizable DOM fraction rather than absolute DOM concentration [28,75].

### 2.9. Statistical Analysis

**Figure 2:**
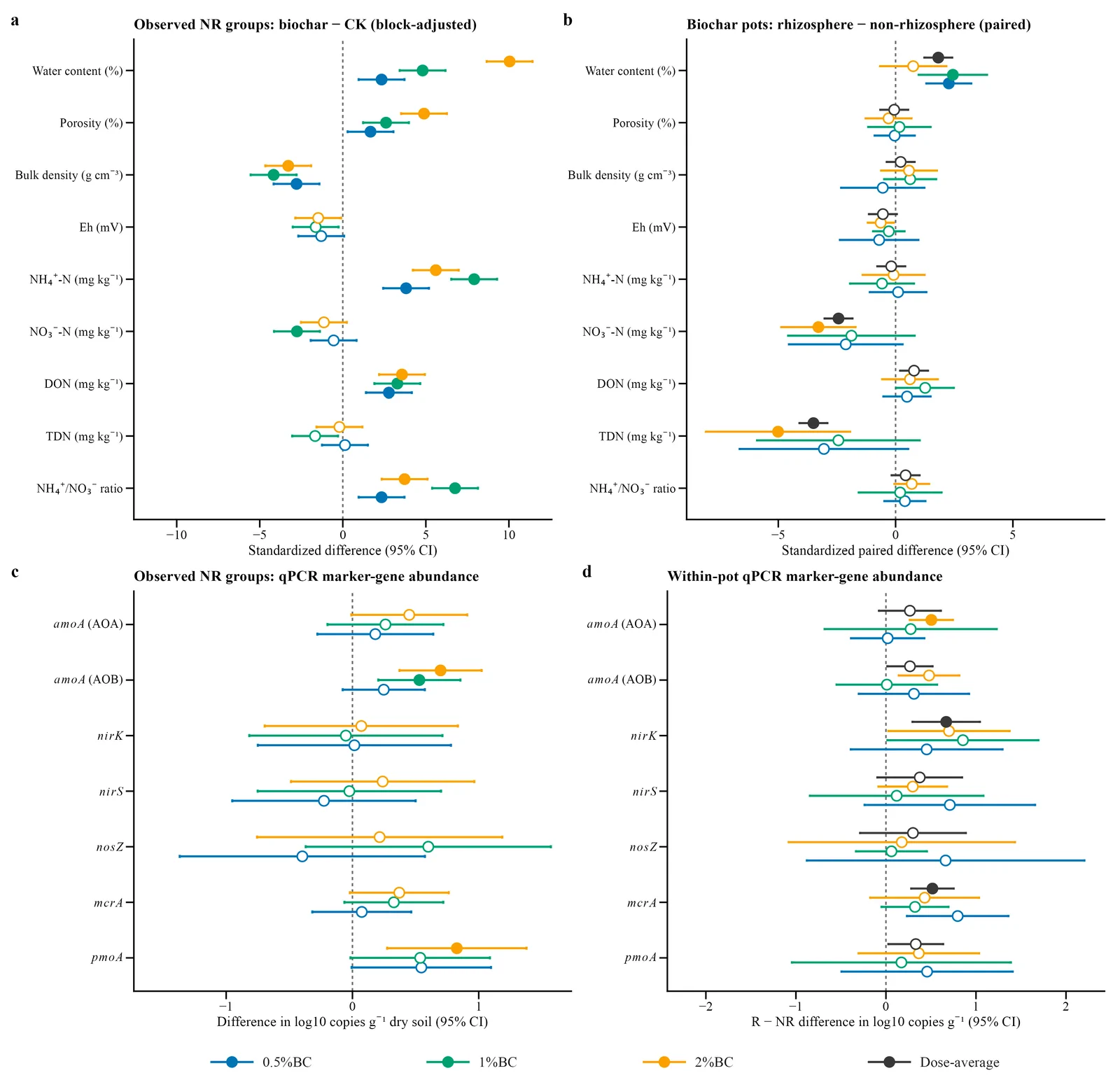
Design-aware soil and qPCR contrasts. (**a**) Block-adjusted differences between each biochar non-rhizosphere (NR) group and CK-NR for soil physical and nitrogen variables. (**b**) Within-pot paired rhizosphere-minus-NR differences for each biochar dose and the equal-weight dose average (grey). Between-pot estimates in panel (**a**) and paired estimates in panel (**b**) are divided by their corresponding model residual SD for display. (**c**) Block-adjusted biochar-NR minus CK-NR differences in log_10_ qPCR copies g^−1^ dry soil. (**d**) Within-pot R−NR qPCR differences and their dose average. Points are estimates and horizontal lines are 95% confidence intervals; filled points indicate Holm-adjusted *p* < 0.05 and open points indicate non-significant Holm-adjusted contrasts within the prespecified contrast family. Blue, green and orange denote 0.5%, 1% and 2% biochar. qPCR measures DNA-level marker-gene abundance, not activity.

Treatment effects in NR soil were estimated from the 20 pots using linear models of the form response ~ block + treatment. The three prespecified biochar-versus-CK contrasts were adjusted within variable using Holm’s method. Within biochar-treated pots, R−NR differences were analyzed as paired samples at each dose (*n* = 5 pairs) and as block-adjusted paired differences across all 15 pots; the four R−NR contrasts were Holm-adjusted within variable. qPCR copy numbers were log_10_-transformed. In Figure 2, between-pot soil estimates were divided by the residual SD from the corresponding block-plus-treatment model, and paired soil estimates by the residual SD from the paired-difference model, solely to place variables on a common visual scale. qPCR panels remain in log_10_ copies g^−1^; all inferential tests were performed in their stated analysis units.

Microbiome inference was divided into identifiable questions. NR treatment effects and biochar-dose effects among R samples were evaluated by PERMANOVA with permutations restricted within blocks. The pooled R−NR effect was tested by swapping zone labels only within pot pairs. Dose-specific R−NR tests used exact within-pair swaps, and dose dependence of the R−NR shift was tested on Hellinger-transformed paired-difference vectors. We requested 9999 permutations; exact permutation sets were used when fewer unique permutations existed. Multivariate dispersion was tested with betadisper and block-restricted permutation [76,77]. PERMANOVA and dispersion were interpreted jointly. FT-ICR MS group plots were descriptive because only three D90 profiles were available per observed group. Treatment-group residual cross-layer associations were FDR-adjusted and regarded as exploratory.

Analyses were performed in R 4.6.0 using vegan 2.7-5, permute 0.9-10 and emmeans 2.0.3; random procedures used seed 20260620.

## 3. Results

### 3.1. Experimental Structure and Inference Boundary

The experiment comprised 20 independent pots in five blocks (Figure 1a). CK maize did not survive, so the five CK pots yielded CK-NR soil only. Each of the 15 biochar-treated pots yielded paired R and NR samples (Figure 1b), giving 35 samples and seven observed sampling groups for the soil, qPCR, 16S and ITS layers; FT-ICR MS used three D90 samples per group (Figure 1c; Table 1). Thus, biochar-versus-CK NR estimates are block-adjusted observed-group contrasts that may include plant-survival effects, while R−NR estimates are within-pot paired contrasts.

### 3.2. Soil Physical Conditions and Nitrogen Pools

Block-adjusted NR comparisons supported a consistent physical pattern (Figure 2a). Relative to CK-NR, water content was higher by 1.72, 3.54 and 7.40 percentage points at 0.5%, 1% and 2% biochar, respectively; porosity was higher by 4.36, 6.80 and 12.78 percentage points; and bulk density was lower by 0.190, 0.284 and 0.224 g cm^−3^ (all Holm-adjusted *p* < 0.05). Eh did not remain significant after adjustment. Within biochar pots, paired R soil had higher water content at 0.5% and 1% and across the dose-averaged contrast (+4.22 percentage points, Holm-adjusted *p* < 0.001), whereas porosity and bulk density showed no adjusted R−NR difference (Figure 2b).

NH_4_^+^–N, DON and the NH_4_^+^/NO_3_^−^ ratio were higher in all three biochar-NR groups than in CK-NR (Holm-adjusted *p* < 0.01), while NO_3_^−^–N was lower only for 1% biochar (−84.3 mg kg^−1^, *p* = 0.0028). The dose-averaged paired R−NR contrast showed lower NO_3_^−^–N (−43.37 mg kg^−1^) and TDN (−37.48 mg kg^−1^; both *p* < 0.001), driven most clearly by the 2% treatment. These results indicate altered N-pool partitioning rather than a uniform increase in dissolved N.

### 3.3. DNA-Level Marker-Gene Abundance

Among block-adjusted NR contrasts, bacterial *amoA* abundance was higher at 1% (+0.530 log_10_ copies g^−1^, *p* = 0.0080) and 2% biochar (+0.698, *p* = 0.0016), and *pmoA* was higher at 2% (+0.827, *p* = 0.0203) than in CK-NR (Figure 2c). Other dose-specific NR marker-gene contrasts did not pass their Holm-adjusted tests. In paired samples, archaeal *amoA* was higher in 2% R than 2% NR (+0.504 log_10_ copies g^−1^, 95% CI 0.265–0.744; *p* = 0.0171). Across doses, mean R−NR differences were positive for *nirK* (+0.669, *p* = 0.0134) and *mcrA* (+0.516 log_10_ copies g^−1^, *p* = 0.0039; Figure 2d). These are changes in DNA-target abundance, not measured nitrification, denitrification, methanogenesis or methane oxidation.

### 3.4. Bacterial and Fungal Community Structure

**Figure 3:**
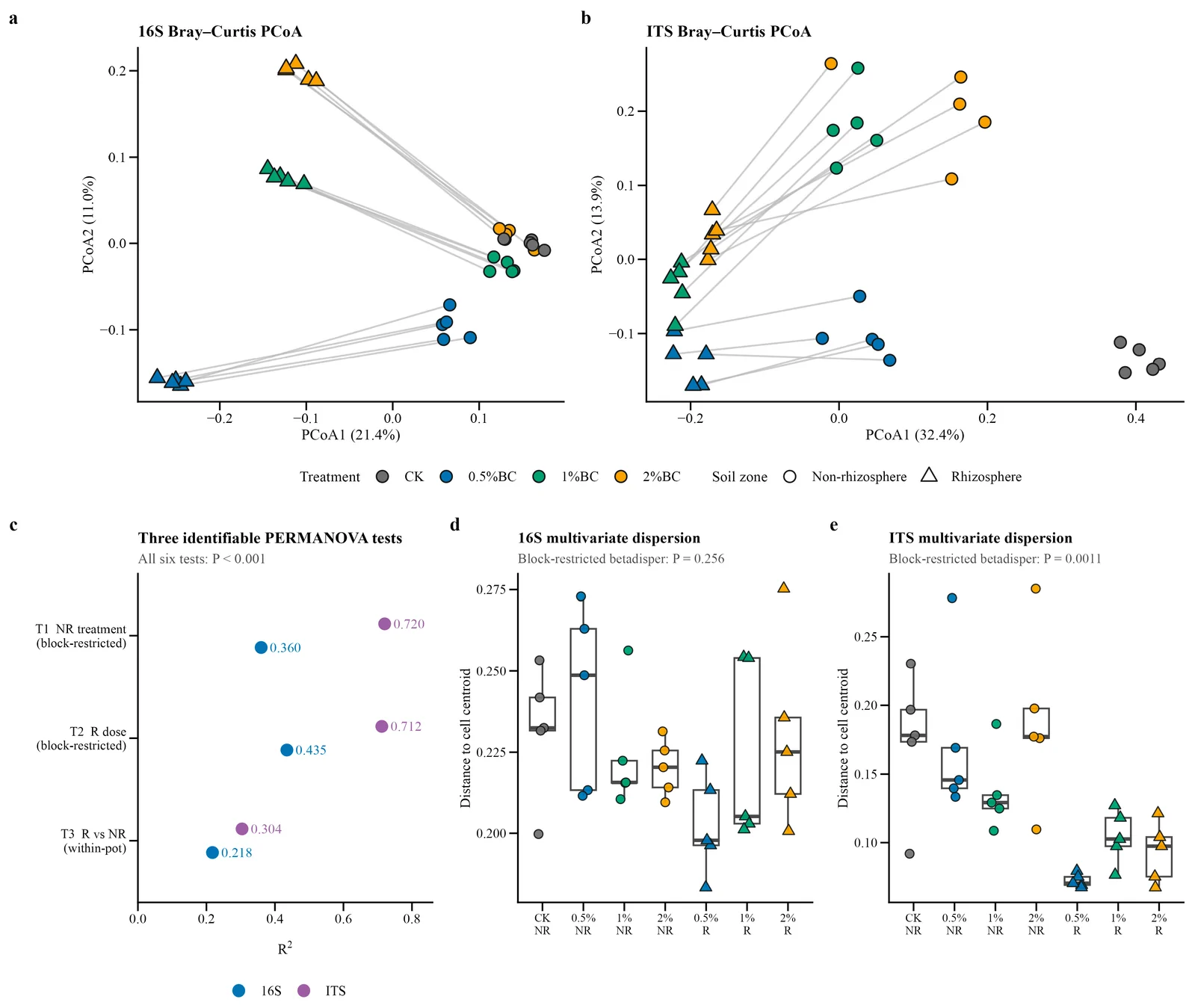
Design-aware bacterial and fungal community structure. (**a**,**b**) Bray–Curtis PCoA for rarefied 16S and ITS profiles. Color denotes treatment, circles denote NR and triangles denote R; grey lines connect paired R/NR samples from the same biochar-treated pot. CK has NR samples only. (**c**) R^2^ for the three identifiable block- or pot-restricted PERMANOVA tests: NR treatment, biochar dose among R samples and the pooled within-pot R−NR contrast. All six tests had *p* < 0.001. (**d**,**e**) Distances to the group centroid for 16S and ITS with block-restricted betadisper *p* values. The significant ITS dispersion test means that ITS PERMANOVA differentiation includes changes in within-group spread and cannot be interpreted solely as a uniform centroid shift [76,77].

Design-aware Bray–Curtis tests detected community differentiation at each identifiable level (Figure 3). For 16S, NR treatment, R-sample biochar dose and the pooled paired R−NR contrast explained 36.0%, 43.5% and 21.8% of variation, respectively (all *p* < 0.001). Corresponding ITS values were 72.0%, 71.2% and 30.4% (all *p* < 0.001). At each individual biochar dose, the exact paired test was limited to 32 label configurations and returned *p* = 0.0625 for both markers; these dose-specific separations were therefore not described as independently significant. The paired compartment-shift vector differed among doses (16S R^2^ = 0.213; ITS R^2^ = 0.521; both *p* < 0.001).

Multivariate dispersion did not differ detectably among the seven observed 16S groups (block-restricted *p* = 0.256), but did differ for ITS (*p* = 0.0011; Figure 3d,e). The fungal PERMANOVA results therefore contain both group-location and within-group dispersion components. Taxonomic profiles and Kruskal–Wallis candidate-genera screens are reported in Figures S2–S4 as descriptive or screening-level outputs, not as confirmed differential taxa.

### 3.5. Relative DOM Profiles and Cross-Layer Associations

**Figure 4:**
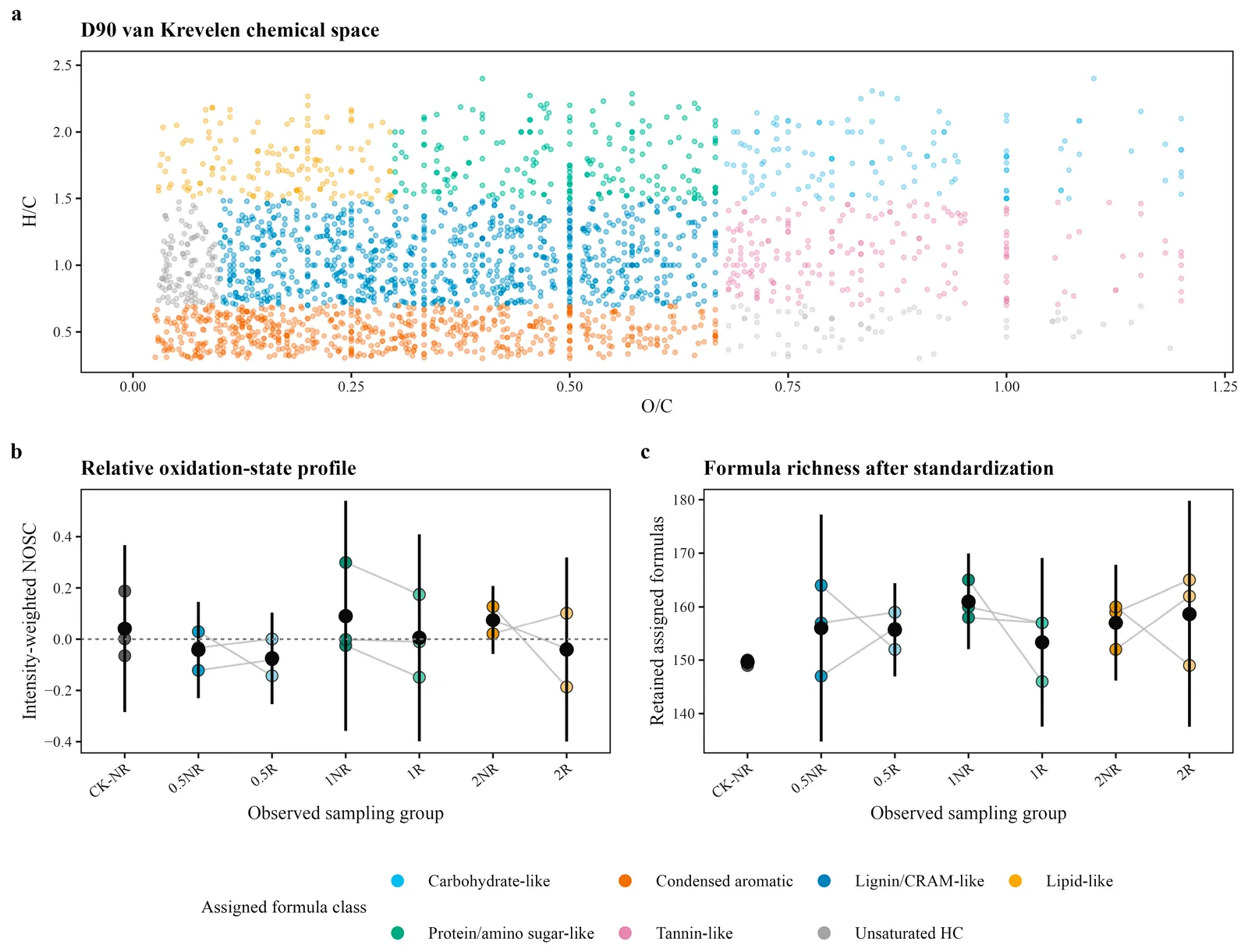
Relative D90 FT-ICR MS DOM molecular profiles. (**a**) Van Krevelen space of retained assigned formulas, colored by formula class. Points represent detected formulas in the D90 profiles and do not represent absolute DOM concentrations. (**b**) Intensity-weighted nominal oxidation state of carbon (NOSC). (**c**) Number of retained formulas after within-sample standardization. In panels (**b**,**c**), colored points are individual profiles, black points and bars are group means and 95% confidence intervals, and grey lines join R/NR profiles sharing a biochar dose and replicate identifier. CK-NR was unpaired; *n* = 3 per group. Panels (**b**,**c**) are descriptive because of the limited D90 replication.

D90 assigned formulas occupied a broad van Krevelen space, and normalized weighted NOSC and formula richness varied among individual profiles (Figure 4). Paired trajectories did not show a consistent dose-wide R−NR direction, and this layer was retained as descriptive because only three profiles were available per observed group. Compound-class summaries are shown in Figure S11. All peak intensities are within-sample relative signals and do not quantify absolute DOM concentration.

Exploratory treatment-group residual associations among soil, qPCR, microbiome and DOM variables are provided in Figure S5; none remained significant after false-discovery rate correction. Assembly, co-abundance and annotation-based functional summaries were also retained in the Supplementary Materials (Figures S7–S10) as exploratory outputs. They were not used as evidence of a causal cross-layer mechanism.

## 4. Discussion

The revised analysis resolves two design features that materially change interpretation: treatment was randomized among 20 pots in five blocks, and R/NR samples were paired subsamples rather than independent experimental units. Within that structure, the clearest pattern was a biochar-associated change in the measured physical habitat and N-pool partitioning, accompanied by selected marker-gene and community responses. The results meet the objective of integrating these measured layers, but they do not isolate a pure biochar mechanism because CK maize did not survive.

All three biochar-NR groups had higher water content and porosity and lower bulk density than CK-NR. These directions agree with broader syntheses of biochar in salt-affected soil, while the magnitude of response is known to depend on soil salinity, biochar properties, dose and crop setting [11,13,14,15,16,78]. The within-pot result—higher R water content at 0.5% and 1% but no consistent paired porosity or bulk-density shift—also shows that the rhizosphere did not simply reproduce the NR dose pattern. However, CK-NR came from pots without surviving plants, whereas biochar-NR came from planted pots with surviving maize. The NR contrasts therefore support differences among the observed pot groups, not a treatment-only estimate independent of plant survival.

The N results point to redistribution among pools rather than generalized N enrichment. A coastal saline-soil experiment using a comparable biochar range reported reduced N leaching and greater N retention at appropriate rates [79], and biochar surfaces, aeration and water status can jointly influence mineral-N fate [11,13,14,15,16,24,25,26]. In our experiment, leaching, gross nitrification and denitrification were not measured, so those processes remain alternatives rather than demonstrated explanations. The qPCR data sharpen this boundary: higher bacterial *amoA* and *pmoA* in selected NR contrasts and positive paired *nirK* and *mcrA* responses identify changes in carrier populations or genetic potential. Cross-study evidence shows that protein-coding gene abundance commonly corresponds only weakly to process rates [48]; our copy-number patterns therefore cannot be read as measured N or methane flux.

Bacterial and fungal communities both differentiated across the identifiable treatment, dose and pooled compartment tests. The paired PCoA structure is consistent with strong root-associated filtering reported in maize and other crops [6,7,8,19,20,21], but the exact dose-specific tests illustrate the limited resolution of five pairs: *p* = 0.0625 is the smallest attainable two-sided separation under the implemented label swaps and should not be interpreted as proof of no compartment effect. The fungal result requires an additional qualification. Distance-based tests can respond to both group centroids and dispersion [76,77]; because ITS dispersion differed, the fungal pattern reflects group differentiation that includes heterogeneous within-group variability. Candidate genera in the supplement are consequently priorities for independent validation, not biomarkers established by this experiment.

FT-ICR MS supplied a molecular context for the soil and microbiome layers, not a concentration-calibrated measure of carbon availability. Electrospray response depends on molecular composition and instrument conditions, so normalized intensities represent the detectable ionizable fraction [27,28,29,30,71]. The absence of FDR-significant residual associations is therefore informative: the parallel soil, gene, community and DOM patterns did not provide statistical support for a specific cross-layer pathway. Time-resolved DOM, transcript or enzyme measurements and direct C/N process rates would be needed to test the proposed links.

The practical contribution is an indication, within the bounds of the study design, that 0.5–2% maize–straw biochar was associated with altered water, pore and N conditions in these salt-affected pots and that the microbial community did not respond as a single, uniform endpoint. This may help prioritize doses and process measurements for a balanced validation experiment, but it is not evidence of yield improvement or field-scale reclamation. The main limitations are CK plant failure, *n* = 5 pots per treatment, only three D90 DOM profiles per group and no direct process-rate measurements. Future work should include a viable planted control, an unplanted factorial control, balanced compartment sampling, repeated harvests, crop performance, absolute DOM and mineral-N budgets, and activity-level microbial measurements.

## 5. Conclusions

In this 90-day blocked pot experiment, biochar-associated differences in soil water, pore structure and nitrogen partitioning were accompanied by selected DNA-level marker-gene responses, bacterial and fungal community differentiation and variation in relative DOM molecular profiles. The R/NR evidence was supported by within-pot pairing, whereas CK-NR versus biochar-NR comparisons were confounded by the absence versus survival of maize. ITS differentiation also included heterogeneous dispersion, and no cross-layer residual association survived FDR correction. The findings therefore identify testable soil-process patterns under the tested pot conditions, not a pure causal biochar mechanism or demonstrated improvement in maize production.

## Figures and Tables

**Table 1:** Observed sampling groups, replication and statistical-unit structure. CK-R did not exist because maize did not survive in CK pots.

Evidence Layer	Observed Samples	Experimental Unit or Pairing	Role in Interpretation
Soil physical and nitrogen	Seven groups; *n* = 5 per group	20 pots; R/NR paired in 15 biochar pots	Habitat and N-pool status
qPCR	Seven groups; *n* = 5 per group	Same 20-pot structure; R/NR paired	DNA-level marker-gene abundance
16S microbiome	Seven groups; *n* = 5 per group	Same 20-pot structure; R/NR paired	Bacterial community structure
ITS microbiome	Seven groups; *n* = 5 per group	Same 20-pot structure; R/NR paired	Fungal community structure and dispersion
FT-ICR MS D90	Seven groups; *n* = 3 per group	Three replicate identifiers per group; paired R/NR in biochar groups	Relative DOM molecular profiles

## Data Availability

Raw 16S and ITS reads have been deposited in the Genome Sequence Archive under BioProject PRJCA066142 and submission subCRA072425. Processed feature tables, qPCR source data, source data for revised main Figure 1, Figure 2, Figure 3 and Figure 4 and selected Supplementary figures, and the design-aware R analysis script are provided with this revision.

## Supplementary Materials

The following supporting information can be downloaded at: https://www.mdpi.com/article/10.3390/microorganisms14091907/s1, Supplementary methods, Tables S1–S11, Figures S1–S11, qPCR source data, design-aware statistical outputs and an ancillary pot-level-derived greenhouse-gas archive are provided with this revision. The greenhouse-gas archive is retained for provenance only and is not part of the manuscript’s numerical synthesis.
