# Supplementary material for: Biochar-Associated Shifts in Nitrogen Status, Microbial Communities and Dissolved Organic Matter in Salt-Affected Maize Soil
Source: Microorganisms. 2026 Aug 28;14(9):1907. doi: 10.3390/microorganisms14091907 (PMC13609317; doi:10.3390/microorganisms14091907)
Supplement: Supplementary file 1 [file microorganisms-14-01907-s001.zip › microorganisms-4523273-supplementary.pdf]

Supplementary material for

Biochar-associated shifts in nitrogen status, microbial communities and dissolved organic matter in salt-affected maize soil

Rui Li [1,2,3*], Chi Zhang [1], Yu Miao [1], Fangze Li [1], Ge Zhang [1], Qiwei Sun [1], Tianci Hua [1], Zhikun Pang [1] and Xingjie Lin [1,4]

[1] BGRIMM Technology Group Co., Ltd., Beijing 100160, China.
[2] Research Center for Eco-Environmental Sciences, Chinese Academy of Sciences, Beijing 100085, China.
[3] University of Chinese Academy of Sciences, Beijing 100049, China.
[4] University of Science and Technology Beijing, Beijing 100083, China.
* Correspondence: lirui18@mails.ucas.edu.cn (R.L.).

Supplementary Methods

S1.1. Pot experiment, management and D90 sampling

Salt-affected soil from the Yellow River Delta (initial pH 8.83; electrical conductivity 2.41 dS m^{-1}) was air-dried, passed through a 2-mm sieve and placed at 8 kg per pot. Four whole-pot biochar rates were used: CK, 0.5%, 1% and 2% (w/w), corresponding to 0, 5, 10 and 20 g kg^{-1} and 0, 40, 80 and 160 g pot^{-1}. The randomized complete block design contained five blocks and one pot per treatment in each block (20 independent pots). Three seeds of *Zea mays* L. cv. Zhengdan 958 were sown per pot on 11 April 2023 and thinned to one plant at V3. Pots were maintained at 25 ± 3 °C under natural light, irrigated gravimetrically with tap water to 60–70% water-holding capacity and received no mineral fertilizer, insecticide or fungicide.

Destructive sampling was conducted on 10 July 2023 (D90). Maize did not survive in the five CK pots, which therefore yielded non-rhizosphere soil (CK-NR) only. Each of the 15 biochar-treated pots yielded a paired rhizosphere (R) sample, defined as soil remaining attached to roots after gentle shaking, and a non-rhizosphere (NR) sample collected more than 1 cm from roots. Treatment × block was used as the pot identifier; R and NR from the same pot were paired subsamples, not independent treatment replicates. Approximately 500 g per soil sample was sieved and partitioned for −80 °C storage (DNA), 4 °C assays or air-dried analyses. CK-NR versus biochar-NR estimates are observed-group or biochar-associated contrasts that can include plant-survival and plant-mediated effects.

S1.2. DNA extraction and quality control

DNA was extracted from 0.5 g fresh soil with the E.Z.N.A. Soil DNA Kit (D5625-02; Omega Bio-tek, Norcross, GA, USA). Soil was bead-beaten for 4 min in 1 mL SLX-Mlus buffer, treated with 100 µL P2 buffer, incubated on ice for 5 min and centrifuged at 13,000 × g for 1 min. The supernatant was treated with 100 µL cHTR reagent, mixed with an equal volume of XP1 buffer, loaded onto a HiBind DNA Mini Column, washed once with 500 µL HBC buffer and twice with 700 µL DNA Wash Buffer, and dry-spun for 2 min. DNA was eluted after adding 70 µL buffer preheated to 70 °C; the recovered volume used in qPCR calculations was approximately 65 µL.

Extracts were quantified with Qubit 3.0, assessed by NanoDrop absorbance and inspected on 1.2% agarose gels. Recorded concentrations were 28–116 ng μL^{-1}, A260/280 was 1.80–2.05 and A260/230 was 1.20–1.90. The 35 samples were processed in two extraction batches (18 and 17 samples), each with one extraction blank. Both blanks contained <0.1 ng μL^{-1} DNA, showed no qPCR amplification and were not sequenced. The same DNA extracts were used for qPCR and 16S/ITS library preparation.

S1.3. qPCR marker-gene assays

Seven DNA-level marker genes were quantified: archaeal and bacterial *amoA*, *nirK*, *nirS*, *nosZ*, *mcrA* and *pmoA*. Reactions were run on a Bio-Rad CFX96 using TB Green Premix Ex Taq II (TaKaRa). Each 20-µL reaction contained 10 µL premix, each primer at 0.4 µM, 1 µL of 1:10-diluted template (approximately 3–12 ng) and nuclease-free water. Cycling was 95 °C for 3 min; 40 cycles of 95 °C for 15 s, the gene-specific annealing temperature for 30 s and 72 °C for 30 s with fluorescence acquisition; followed by a 65–95 °C melt curve in 0.5 °C increments every 5 s. Literal primer sequences, annealing temperatures, expected products and assay performance are given in Table S11.

Each plate contained seven-point, 10-fold plasmid standards spanning 10^1–10^7 copies µL^{-1}, each in triplicate. Target inserts had been cloned into pMD19-T and sequence-verified. Recorded efficiencies were 95.8–101.2% and R^2 values were 0.994–0.999; the limit of detection was 10 copies µL^{-1} and the limit of quantification was 100 copies µL^{-1}. Each sample was run in three technical replicates. If replicate Cq SD was ≥0.5, the plate was repeated once; if the criterion remained exceeded, the median copy number from the two determinations was retained. Three no-template controls per plate and both extraction blanks were undetermined. A 1:10 versus 1:50 dilution check on three samples per plate produced an approximately 2.3-cycle shift.

Copies g^{-1} dry soil were calculated as measured copies µL^{-1} × 10-fold dilution × 65-µL recovered volume divided by the dry-soil mass represented by 0.5 g fresh soil after measured-moisture correction. Values were $\log_{10}$-transformed before modelling. All 14 primers were ordered from Tsingke Biotech on 20 November 2023 under order TS-20231120-0821, PAGE-purified and accepted on 23 November. Fourteen CFX96 plates were run from 5–8 December 2023, two plates per target, with an 18 + 17 sample split, three technical replicates, triplicate seven-point standards, three no-template controls per plate and two extraction blanks. The order records identify FlaCu/R3Cu for *nirK* and cd3aF/R3cd for *nirS*. The literal reverse sequences match Henry-type *nirK*1040R and Kandeler-type *nirS*6QR sequences rather than the sequences originally published under the R3Cu and R3cd names. Table S11 therefore separates supplier-order names from sequence provenance and describes both assays as mixed-source, adapted author protocols.

S1.4. Amplicon library preparation, read processing and provenance

The bacterial 16S rRNA V3–V4 region was amplified with 338F/806R, and the fungal ITS2 region with ITS3/ITS4. Two-step PCR added Nextera-compatible dual indices and independent 12-bp sample barcodes. Illumina bcl2fastq2 v2.20.0 separated orders XQ-20231129-00021 (16S) and XQ-20231129-00024 (ITS) with --barcode-mismatches 0. Cutadapt 4.4 then used the anchored barcode command -g ^{BARCODE} --no-indels --error-rate 0 --discard-untrimmed on the 5′ end of R1; reads without an exact barcode were discarded. After barcode removal, R1 and R2 were oriented to the forward and reverse primer reads. Libraries were quantified with Qubit 4.0, checked with Qsep400 and sequenced by Magigene on an Illumina NovaSeq 6000 in PE250 mode (35 libraries per marker). Library-PCR no-template controls showed no band; the two extraction blanks were not sequenced. Delivered clean-read ranges were 97,098–178,077 for 16S and 98,123–249,880 for ITS, with Q30 ranges of 95.2–95.9% and 94.9–95.7%, respectively.

After barcode removal, fastp 0.23.2 applied a Q20 threshold, removed adapters and retained reads of at least 150 bp. VSEARCH 2.22.1 merged paired ends with --fastq_maxdiffs 15 and --fastq_minovlen 10; ITS additionally used --fastq_allowstaggered. Cutadapt 4.4 trimmed both primers at an error rate of 0.2 and discarded reads lacking the expected primers. VSEARCH 2.22.1 then filtered at --fastq_maxee 1.0 and minimum lengths of 300 bp for 16S and 200 bp for ITS, followed by --derep_fulllength with size annotations and --minuniquesize 2.

UNOISE3 was run in USEARCH v11.0.667 as usearch11 -unoise3 derep.fa -zotus zotus.fa -minsize 8. The command did

not set -unoise_alpha; therefore the USEARCH default alpha of 2.0 applied. UNOISE3 performed abundance-aware error correction and de novo chimera removal to generate zOTUs (exact-sequence features). VSEARCH --uchime3_denovo 2.22.1 provided an additional representative-sequence chimera review, and VSEARCH --usearch_global 2.22.1 mapped filtered reads back to zOTU centroids at ≥99% identity. VSEARCH SINTAX 2.22.1 assigned taxonomy at a confidence cutoff of 0.8 against silva_138.1_ssuref_nr99_sintax.fa for 16S and unite_2022-11-28_sh_refs_dynamic_sintax.fa for ITS. Both were FASTA reference files with hierarchical d:/p:/c:/o:/f:/g:/s: taxonomy paths in the headers; SINTAX used them directly rather than a fitted classifier. Chloroplast and mitochondrial 16S features were removed and only fungal ITS features were retained.

Representative sequences were aligned with MAFFT 7.505 using --auto, trimmed with trimAl 1.4.rev22 using -automated1 and used to construct separate 16S and ITS trees with FastTree 2.1.11 using -nt -gtr. The date embedded in the ITS filename is not treated as the official release date of UNITE 8.3. Alpha-diversity tables used one rarefaction without replacement to 60,000 reads per sample for 16S and 50,000 for ITS (vegan::rrarefy; seed 20260620). Rarefaction curves used nine depths from 1,000 to 70,000 reads with 100 resamplings per depth. Observed richness, Chao1, Shannon, Simpson and Pielou evenness used vegan 2.7-5; Faith's PD used picante 1.8.2 and the corresponding FastTree tree; Good's coverage was retained from the delivered alpha-diversity table. For primary beta-diversity analysis, counts were rarefied to the stated depths, converted to relative abundance and analysed with Bray–Curtis distances; unrarefied relative abundance provided a sensitivity analysis with the same main significance directions.

S1.5. Design-aware statistical analysis

For NR soil, the 20 pots were analysed with response ~ block + treatment and three prespecified biochar-minus-CK contrasts, with Holm adjustment within each variable. Within the 15 biochar pots, R−NR differences were analysed as paired observations at each dose (n = 5 pairs) and as a block-adjusted paired-difference model across doses; three dose-specific contrasts and the equal-weight dose average were Holm-adjusted within variable. qPCR copy numbers were $\log_{10}$-transformed.

Microbiome inference was separated into identifiable questions. NR treatment effects and R-sample biochar-dose effects used PERMANOVA with permutations restricted within blocks. The pooled R−NR effect was tested by swapping zone labels within pot pairs; dose-specific R−NR tests used exact within-pair swaps. Dose dependence of the paired compartment shift was tested on Hellinger-transformed R−NR vectors. We requested 9,999 permutations, using the complete exact set when fewer unique permutations existed. Multivariate dispersion was tested by *betadisper* with block-restricted permutation and interpreted jointly with PERMANOVA. Analyses used R 4.6.0, vegan 2.7-5, permute 0.9-10 and emmeans 2.0.3 with seed 20260620.

S1.6. FT-ICR MS, exploratory outputs and manuscript scope

D90 FT-ICR MS results were based on three profiles per observed group. After blank subtraction, peak lists were standardized to 200 assigned formulas and intensities were normalized within sample. Formula richness, H/C, O/C, DBE, DBE−O, modified aromaticity index, nominal oxidation state of carbon and compound-class profiles therefore describe the detected ionizable DOM fraction, not absolute DOM concentration. D90 group displays are descriptive because the molecular-profile subset had n = 3 per observed group.

Candidate taxa, null-model assembly summaries, CLR co-abundance networks, taxonomic functional profiles and treatment-residual cross-layer associations are exploratory; no residual cross-layer association remained significant after FDR correction. The laboratory reconciliation record maps the legacy 245-row greenhouse-gas tables (35 position-coded

entries × seven dates) to the 20-pot design and provides a 140-row pot-level derived time series by averaging the paired position-coded records within each biochar pot and date while retaining the single CK record. This reconciled archive is retained only as ancillary provenance and remains outside the manuscript's numerical synthesis. The previously supplied cumulative table does not agree exactly with transparent integration of the reconciled time series and is not used as a reproducible cumulative result; an independently recalculated AR6 cumulative archive generated by the documented R workflow is retained only for reproducibility lineage. R/NR-like labels in the legacy files do not create independent gas-measurement units or permit compartment attribution. No greenhouse-gas inference or treatment comparison is made. The raw 16S and ITS reads were deposited under GSA BioProject PRJCA066142/submission subCRA072425, passed repository review and are scheduled for public release upon article acceptance; a final public accession will be added before publication.

Supplementary Tables

Table S1. Biochar feedstock, production conditions and physicochemical properties recorded in the archived source table.

Property	Value	Unit	Method/record note
Feedstock	Corn straw (Zea mays L.)		Collected post-harvest, air-dried, ground <2 mm
Pyrolysis temperature	500	°C	Limited oxygen, heating rate 10°C/min, residence 2 h
Pyrolysis reactor	Muffle furnace (programmable)		N2-purged before heating
Yield	32.5	%	Biochar mass / feedstock mass × 100
pH	9.52		1:10 biochar:water slurry, glass electrode
EC	3.87	dS m^{-1}	1:10 biochar:water extract
TOC	63.14	%	Dry combustion (Vario EL Cube)
TC	63.32	%	Dry combustion
IC	0.18	%	TC - TOC
TN	0.34	%	Kjeldahl digestion
C/N ratio	185		TOC/TN mass ratio
H content	2.87	%	Elemental analyzer (Vario EL Cube)
O content	18.56	%	By difference: 100 - C - H - N - ash (approximate)
Atomic H/C ratio	0.55		H(wt%)/1.008 ÷ C(wt%)/12.011
Atomic O/C ratio	0.22		O(wt%)/16.00 ÷ C(wt%)/12.011
Ash content	16.1	%	550°C muffle furnace, 4 h
Volatile matter	22.3	%	ASTM D1762-84
Fixed carbon	61.6	%	100 - ash - volatile matter
BET surface area	24.8	m^2 g^{-1}	N2 adsorption, BET method (ASAP 2460)
Micropore volume	0.008	cm^3 g^{-1}	t-plot method from N2 adsorption
Total pore volume	0.042	cm^3 g^{-1}	Single point at P/P0 ≈ 0.99

Property	Value	Unit	Method/record note
Average pore diameter	6.8	nm	4V/A by BET
Cation exchange capacity	28.5	cmol kg^{-1}	NH4OAc method at pH 7
Point of zero charge ($pH_{p}z^{c}$)	8.2		pH drift method (point of zero charge)
FTIR characterization	Not available		Not used in interpretation.

Table S2. Verified pot-experiment design, management and sampling boundary.

Item	Verified value or design statement
Soil	Salt-affected Yellow River Delta soil; initial pH 8.83 and EC 2.41 dS m^{-1}
Pot and soil mass	Plastic pot, 20-cm internal diameter and height; drainage holes and tray; 8 kg soil pot^{-1}
Treatments	CK, 0.5%, 1% and 2% biochar (w/w) = 0, 5, 10 and 20 g kg^{-1} = 0, 40, 80 and 160 g pot^{-1}
Design	Randomized complete block design; 5 blocks × 4 treatments = 20 independent pots; one pot per treatment in each block
Experimental unit	The pot. Molecular and soil samples are subsamples of pots, not additional treatment replicates
Crop	*Zea mays* L. cv. Zhengdan 958; three seeds sown, thinned to one plant at V3
Greenhouse	25 ± 3 °C; natural light; gravimetric irrigation to 60–70% water-holding capacity; no mineral fertilizer or pesticide
Timeline	Sown 11 April 2023; destructive harvest and sampling 10 July 2023 (D90)
CK survival	Maize did not survive in any of the five CK pots; CK yielded NR soil only and no true rhizosphere
D90 sampling	Five CK-NR samples plus paired R/NR samples from 15 biochar pots = 35 soil samples
Inference boundary	CK-NR versus biochar-NR is an observed-group/biochar-associated contrast that may include plant-survival and plant-mediated effects

Table S3. Observed sampling groups, replication and dependence structure across evidence layers.

Evidence layer	Observed groups	Biological replication	Unit/dependence	Interpretive role
Soil physical and N	CK-NR; 0.5NR; 1NR; 2NR; 0.5R; 1R; 2R	n = 5 per group	20 pots; R/NR paired within each of 15 biochar pots	Physical habitat and N-pool status
qPCR	Same seven groups	n = 5 per group; 3 technical replicates per assay	Same 20-pot structure; technical replicates averaged by the stated QC rule	DNA-level marker-gene abundance, not process rate
16S	Same seven groups	35 libraries; n = 5 per group	Same 20-pot structure; R/NR paired within pot	Bacterial zOTU community
ITS	Same seven groups	35 libraries; n = 5 per group	Same 20-pot structure; R/NR paired within pot	Fungal zOTU community
FT-ICR MS, D90	Same seven groups	n = 3 profiles per group	Subset; descriptive group profiles	Relative ionizable DOM molecular profile
Greenhouse gases	Four whole-pot treatments; legacy R/NR-like labels are	20 pots × 7 dates = 140 rows in the	Pot is the unit; author-supplied cumulative lineage differs from	Ancillary provenance only; independently recalculated

Evidence layer	Observed groups	Biological replication	Unit/dependence	Interpretive role
	position labels only	reconciled pot-level derived time series	transparent recalculation	AR6 cumulative archive retained for reproducibility lineage; no greenhouse-gas inference

Table S4. qPCR marker-gene panel and the interpretation retained in the manuscript.

Marker gene	Associated functional group/process	Interpretation retained
amoA-AOA	Ammonia-oxidizing archaea; nitrification potential	DNA abundance only; not measured activity or process rate
amoA-AOB	Ammonia-oxidizing bacteria; nitrification potential	DNA abundance only; not measured activity or process rate
nirK	Copper-containing nitrite reductase; denitrification potential	DNA abundance only; not measured activity or process rate
nirS	Cytochrome cd_1 nitrite reductase; denitrification potential	DNA abundance only; not measured activity or process rate
nosZ	Nitrous-oxide reductase; N_2O-reduction potential	DNA abundance only; not measured activity or process rate
mcrA	Methanogenic archaea; methanogenesis potential	DNA abundance only; not measured activity or process rate
pmoA	Methanotrophs; methane-oxidation potential	DNA abundance only; not measured activity or process rate

Table S5. Amplicon targets, delivered read quality, recovered provider processing, normalization and unresolved provenance.

Dataset	Libraries/read QC	Target and primers	Documented processing and taxonomy	Normalization and status
16S	35 PE250 libraries; clean reads 97,098–178,077; Q30 95.2–95.9%	V3–V4; 338F ACTCCTACGGGAGGCAGCAG / 806R GGACTACHVGGGTWTCTAAT	bcl2fastq2 2.20.0 (index mismatch 0); cutadapt 4.4 exact anchored 12-bp R1 barcode; fastp 0.23.2 (Q20; ≥150 bp); VSEARCH 2.22.1 merge (maxdiffs 15; minovlen 10), maxee 1.0/minlen 300 and dereplication; USEARCH 11.0.667 UNOISE3 command with -minsize 8 and default alpha 2.0; VSEARCH uchime3_denovo and ≥99% remap; SINTAX 0.8 with silva_138.1_ssuref_nr99_sintax.fa; MAFFT 7.505; trimAl 1.4.rev22; FastTree 2.1.11 (-nt -gtr)	Single rarefaction to 60,000; rarefaction curves at nine depths with 100 resamplings; primary Bray–Curtis on rarefied relative abundance; unrarefied sensitivity; GSA PRJCA066142/subCRA072425
ITS	35 PE250 libraries; clean reads 98,123–249,880; Q30 94.9–95.7%	ITS2; ITS3 GCATCGATGAAGAACGCAGC / ITS4 TCCTCCGCTTATTGATATGC	bcl2fastq2 2.20.0 (index mismatch 0); cutadapt 4.4 exact anchored 12-bp R1 barcode; fastp 0.23.2; VSEARCH 2.22.1 merge (maxdiffs 15; minovlen 10; allowstaggered), maxee 1.0/minlen 200 and dereplication; USEARCH 11.0.667 UNOISE3 command with -minsize 8 and default alpha 2.0; VSEARCH uchime3_denovo and ≥99% remap; SINTAX 0.8 with unite_2022-11-28_sh_refs_dynamic_sintax.fa; MAFFT 7.505; trimAl	Single rarefaction to 50,000; rarefaction curves at nine depths with 100 resamplings; primary Bray–Curtis on rarefied relative abundance; unrarefied sensitivity; GSA PRJCA066142/subCRA072425

Dataset	Libraries/read QC	Target and primers	Documented processing and taxonomy	Normalization and status
			1.4.rev22; FastTree 2.1.11 (-nt -gtr)	
Documentary boundary	—	—	Literal fastp command and exact reference FASTA files not archived; later author statement supplies valid-format MD5/SHA-256 strings, but the digests were not independently recomputed. The ITS filename date is not treated as an official UNITE release date	Workflow settings are author-supplied; local vendor reports independently confirm barcode-oriented preprocessing and read-quality summaries, not the complete downstream command history

Table S6. Design-aware PERMANOVA and multivariate-dispersion summaries for bacterial and fungal communities. Primary results used rarefied relative abundance and Bray–Curtis distance unless Hellinger vectors are specified.

Marker	Design-aware test	Permutation restriction	R^2	P	Interpretation
16S	NR treatment effect	Within block	0.360	<0.001	Block-aware between-pot treatment contrast
16S	R-sample biochar-dose effect	Within block	0.435	<0.001	Block-aware dose contrast among R samples
16S	Pooled R−NR effect	Zone labels swapped within each of 15 pot pairs	0.218	<0.001	Paired compartment effect
16S	Dose-specific R−NR effects (0.5%, 1%, 2%)	Exact swaps within five pairs per dose	0.425; 0.352; 0.374	0.0625 each	Minimum attainable two-sided resolution with five pairs; not dose-wise confirmation
16S	Dose dependence of paired shift	Within-block permutations of R−NR Hellinger vectors	0.213	<0.001	Compartment-shift vectors differed among doses
16S	Seven-group dispersion	Block-restricted *betadisper* permutation	—	0.2563	No detected dispersion heterogeneity
ITS	NR treatment effect	Within block	0.720	<0.001	Block-aware between-pot treatment contrast
ITS	R-sample biochar-dose effect	Within block	0.712	<0.001	Block-aware dose contrast among R samples
ITS	Pooled R−NR effect	Zone labels swapped within each of 15 pot pairs	0.304	<0.001	Paired compartment effect
ITS	Dose-specific R−NR effects (0.5%, 1%, 2%)	Exact swaps within five pairs per dose	0.606; 0.734; 0.661	0.0625 each	Minimum attainable two-sided resolution with five pairs; not dose-wise confirmation
ITS	Dose dependence of paired shift	Within-block permutations of R−NR Hellinger vectors	0.521	<0.001	Compartment-shift vectors differed among doses
ITS	Seven-group dispersion	Block-restricted *betadisper*	—	0.0011	PERMANOVA may reflect centroid and

Marke r	Design-aware test	Permutation restriction	R^2	P	Interpretation
		permutation			dispersion differences; interpret jointly

Table S7. Representative screening-level candidate genera reported in the main manuscript.

Dataset	Representative candidates	Screening method	Evidence boundary
16S	Echinicola; Ensifer; Chitinophaga	Kruskal–Wallis screening with FDR adjustment	Candidate indicators only
ITS	Metarhizium; Aspergillus; Penicillium	Kruskal–Wallis screening with FDR adjustment	Candidate indicators only

Table S8. FT-ICR MS acquisition, formula-assignment and filtering criteria used for D90 DOM profiles.

Stage	Criterion or record
Extraction	Milli-Q water extraction followed by solid-phase extraction
Instrument	Bruker SolariX 12-T Fourier-transform ion cyclotron resonance mass spectrometer; negative-mode electrospray ionisation
Acquisition range	m/z 150–1,200
Initial assignment	Monoisotopic peaks with S/N ≥ 4; $C_{1–60}H_{1–120}O_{1–30}N_{0–5}S_{0–3}$; mass error < 1.0 ppm; $[M−H]^−$ ions
Chemical plausibility	0.3 ≤ H/C ≤ 2.5; 0 ≤ O/C ≤ 1.2; integer DBE and valence rules
Final signal threshold	S/N ≥ 6
Homologous-series filter	CH_2 series with at least three members; Kendrick mass-defect tolerance ± 0.001
Standardization	Blank subtraction followed by standardization to 200 assigned formulas per sample
Analytical subset	D90 profiles; n = 3 per observed group
Intensity interpretation	Within-sample normalized relative signal, not absolute concentration

Table S9. DOM molecular metrics, compound classes and interpretation boundaries.

Metric or class	Use in this study	Interpretation boundary
Formula richness	Number of retained assigned formulas	Relative profile after standardized filtering
H/C and O/C	Van Krevelen molecular-space descriptors	Formula-level molecular context
DBE and DBE−O	Unsaturation and oxygen-adjusted unsaturation descriptors	Calculated from assigned formulas
AI_mod	Modified aromaticity index	Directional molecular-trait descriptor
NOSC	Nominal oxidation state of carbon	Directional molecular-trait descriptor
Compound classes	Carbohydrate-like, condensed aromatic, lignin/CRAM-like, lipid-like, protein/amino-sugar-like, tannin-like and others	Percentage of within-sample normalized FT-ICR MS signal

Table S10. Interpretation rules for exploratory association, network, assembly and functional-profile outputs.

Analysis layer	Displayed output	Boundary retained
Treatment-controlled residual associations	Nominal microbe–soil–qPCR–DOM association matrix	No association survived FDR correction; hypothesis-generating only
Co-abundance network	Global CLR network with positive and negative edges	Associations, not direct microbial interactions
Module–environment coupling	Nominal module associations and leading taxa	Treatment-structured descriptive patterns
Community assembly	Null-model process categories	Null-model-inferred exploratory summary
Functional profiles	Taxonomy-derived bacterial functions and fungal guilds	Putative annotations, not measured activity
Greenhouse-gas variables	Reconciled 140-row pot-level time series; author-supplied cumulative lineage differs from transparent recalculation	Ancillary only; independently recalculated AR6 cumulative archive retained; no greenhouse-gas inference

Table S11. qPCR primer sequences, annealing temperatures, expected products, standard-curve performance and source audit.

Target	Forward primer and literal sequence (5′→3′)	Reverse primer and literal sequence (5′→3′)	Ta (°C)	Product	Slope; efficiency; R^2	Source and sequence-status note
AOA *amoA*	Arch-*amoA*F STAATGGTCTGGCTTAGACG	Arch-*amoA*R GCGGCCATCCATCTGTATGT	55	~635 bp	−3.35; 99.1%; 0.998	Francis et al. (2005); exact sequence match
AOB *amoA*	*amoA*-1F GGGGTTTCTACTGGTGGT	*amoA*-2R CCCCTCKGSAAAGCCTTCTTC	58	~491 bp	−3.38; 97.8%; 0.996	Rotthauwe et al. (1997); exact sequence match
nirK	Supplier-order FlaCu ATCATGGTSCTGCCGCG	Supplier-order R3Cu GCCTCGATCAGRTTRTGGTT	57	~473 bp	−3.36; 98.5%; 0.997	Laboratory/supplier-order reconciliation record identifies FlaCu/R3Cu. Literal sequence provenance is mixed-source (Hallin-type forward + Henry *nirK*1040R-type reverse); adapted author protocol, not the original published R3Cu pair
nirS	Supplier-order cd3aF GTSAACGTSAAGGARACSGG	Supplier-order R3cd GASTTCGGRTGSGTCTTSAYGAA	57	~425 bp	−3.40; 96.9%; 0.995	Laboratory/supplier-order reconciliation record identifies cd3aF/R3cd. Literal sequence provenance is mixed-source (Throbäck cd3aF-type forward + Kandeler *nirS*6QR-type reverse); adapted author protocol, not the original published R3cd pair
nosZ	*nosZ*-F CGYTGTTCMTCGACAGCCAG	*nosZ*-R CATGTGCAGNGCRTGGCAGAA	60	~700 bp	−3.43; 95.8%; 0.994	Rösch et al. (2002); exact sequence match
mcrA	mlas GGTGGTGTMGGDTTCACMCARTA	*mcrA*-rev CGTTCATBGCGTAGTTVGGRTAGT	55	~470 bp	−3.29; 101.2%; 0.999	Steinberg and Regan (2008); exact sequence match
pmoA	A189F GGNGACTGGGACTTCTGG	mb661R CCGGMGCAACGTCYTTACC	55	~471 bp	−3.39; 97.2%; 0.997	Costello and Lidstrom (1999); exact sequence match; F/R suffixes are directional labels

Table S11 assay-wide conditions. Reaction volume was 20 µL (10 µL TB Green Premix Ex Taq II, each primer at 0.4 µM,

1 µL 1:10-diluted template and nuclease-free water). Cycling was 95 °C for 3 min; 40 × (95 °C for 15 s, target-specific Ta for 30 s, 72 °C for 30 s); and a 65–95 °C melt curve. Seven plasmid standards (10^1–10^7 copies µL^{-1}) and all samples were run in triplicate. LOD and LOQ were 10 and 100 copies µL^{-1}. Three NTCs per plate and two extraction blanks were undetermined. Copy-number conversion used the 10-fold dilution, 65-µL recovered extract volume and moisture-corrected dry mass represented by 0.5 g fresh soil.

Supplementary Figures

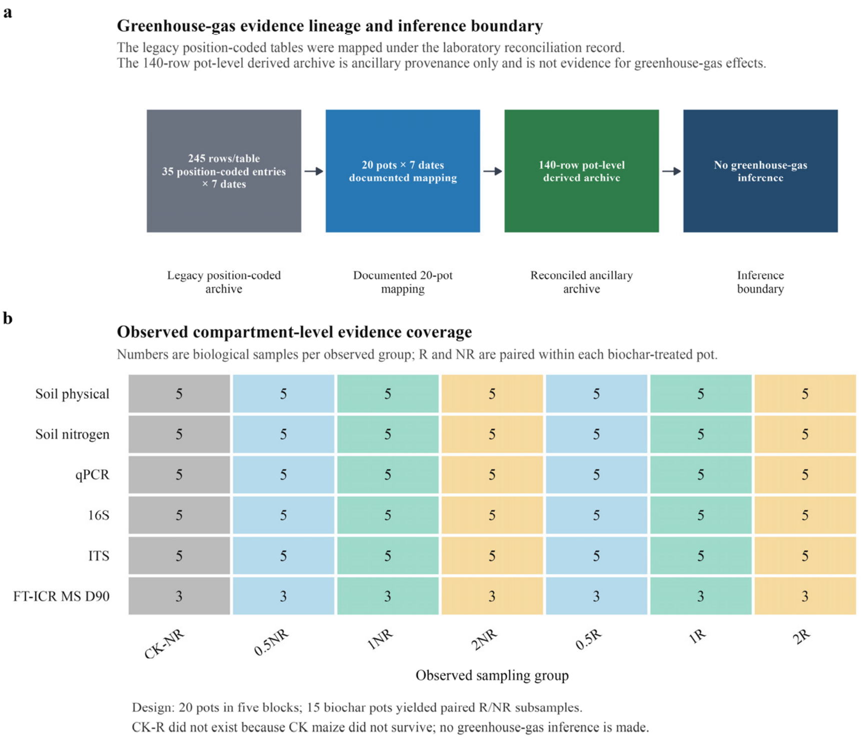

	CK-NR	0.5NR	1NR	2NR	0.5R	1R	2R
Soil physical	5	5	5	5	5	5	5
Soil nitrogen	5	5	5	5	5	5	5
qPCR	5	5	5	5	5	5	5
16S	5	5	5	5	5	5	5
ITS	5	5	5	5	5	5	5
FT-ICR MS D90	3	3	3	3	3	3	3

Figure S1. Evidence lineage, coverage and manuscript scope. (A) The legacy greenhouse-gas tables contain 245 position-coded rows per table. The documented 20-pot mapping produced a 140-row pot-level derived time series (20 pots × seven dates). The previously supplied cumulative table differs from transparent integration of that time series; an independently recalculated AR6 cumulative archive is therefore retained for reproducibility lineage. Both cumulative files and the reconciled time series are ancillary provenance only, outside the numerical synthesis, and no greenhouse-gas inference is made. (B) Coverage of the seven observed soil groups and evidence layers from 20 pots, including 15 within-pot R/NR pairs. CK-NR came from pots without surviving maize.

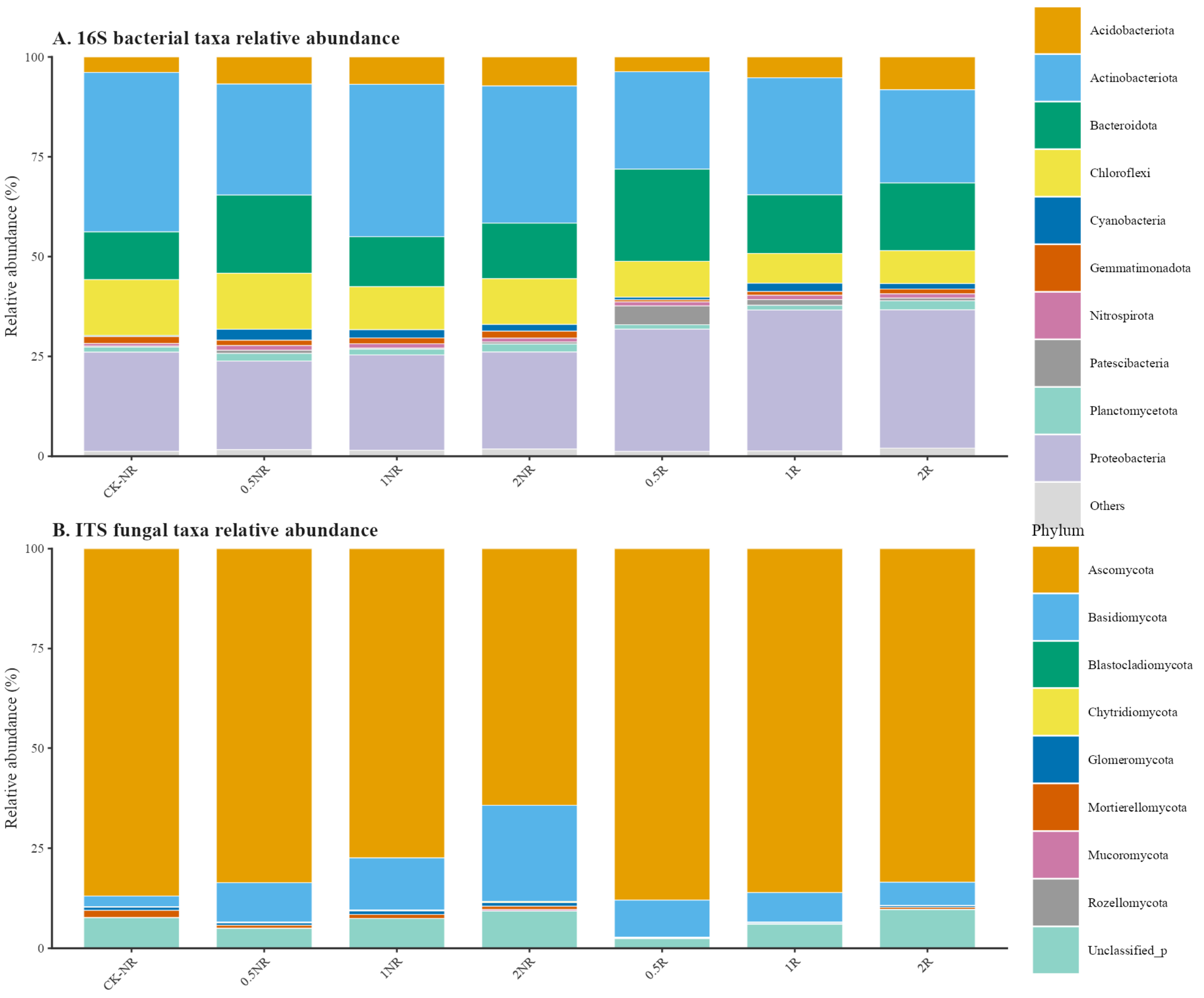

Figure S2. Phylum-level bacterial and fungal community profiles across the seven observed groups. (A) Mean relative abundance of displayed 16S bacterial phyla. (B) Mean relative abundance of displayed ITS fungal phyla. The panels are descriptive compositions of UNOISE3-derived zOTUs (exact-sequence features), not independent differential-abundance tests.

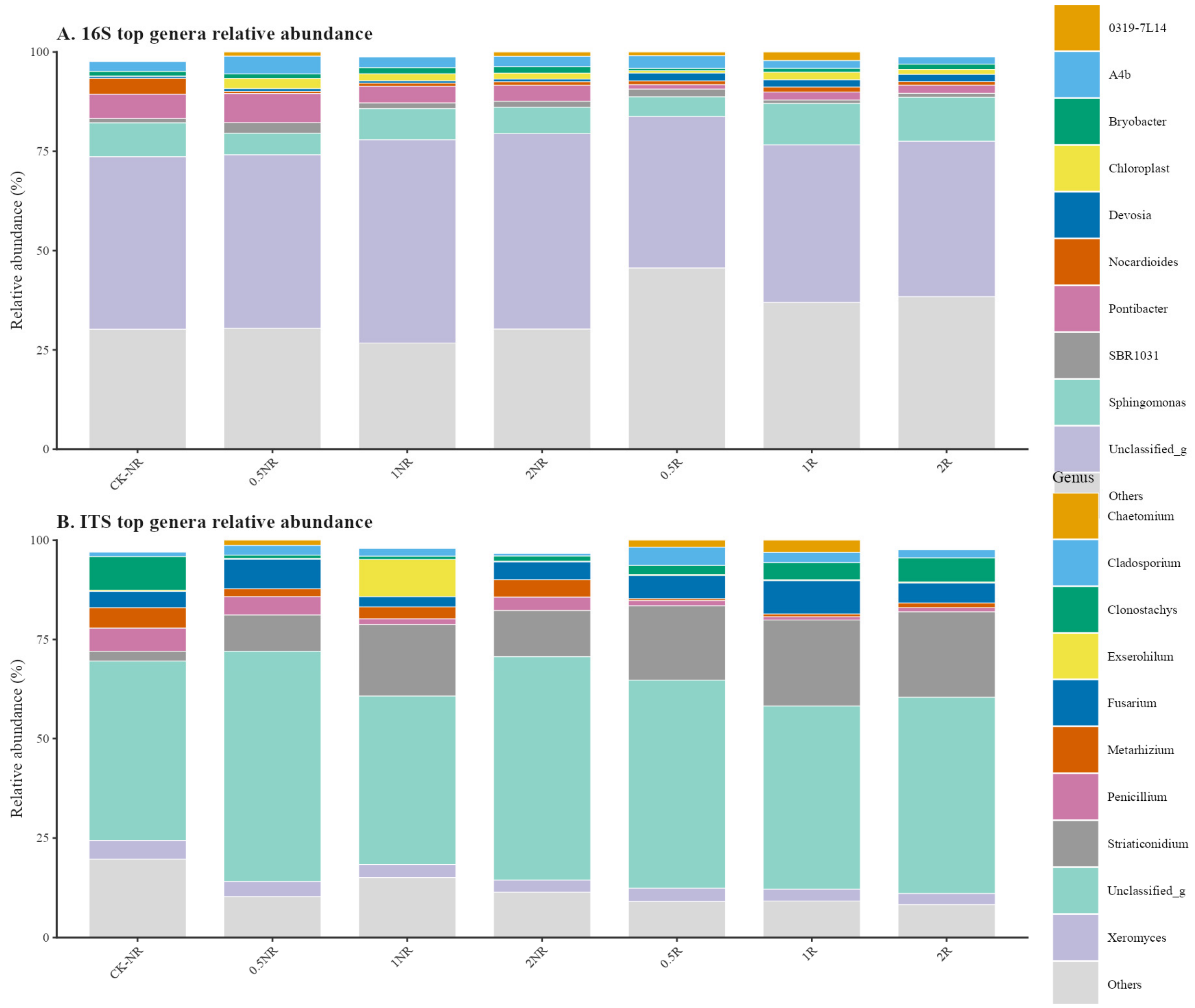

Figure S3. Profiles of the most abundant bacterial genera and fungal taxa across the seven observed groups. (A) Group-mean relative abundance of displayed 16S genera. (B) Group-mean relative abundance of displayed ITS taxa. Lower-abundance assignments are included in the aggregated remainder; the panels are descriptive.

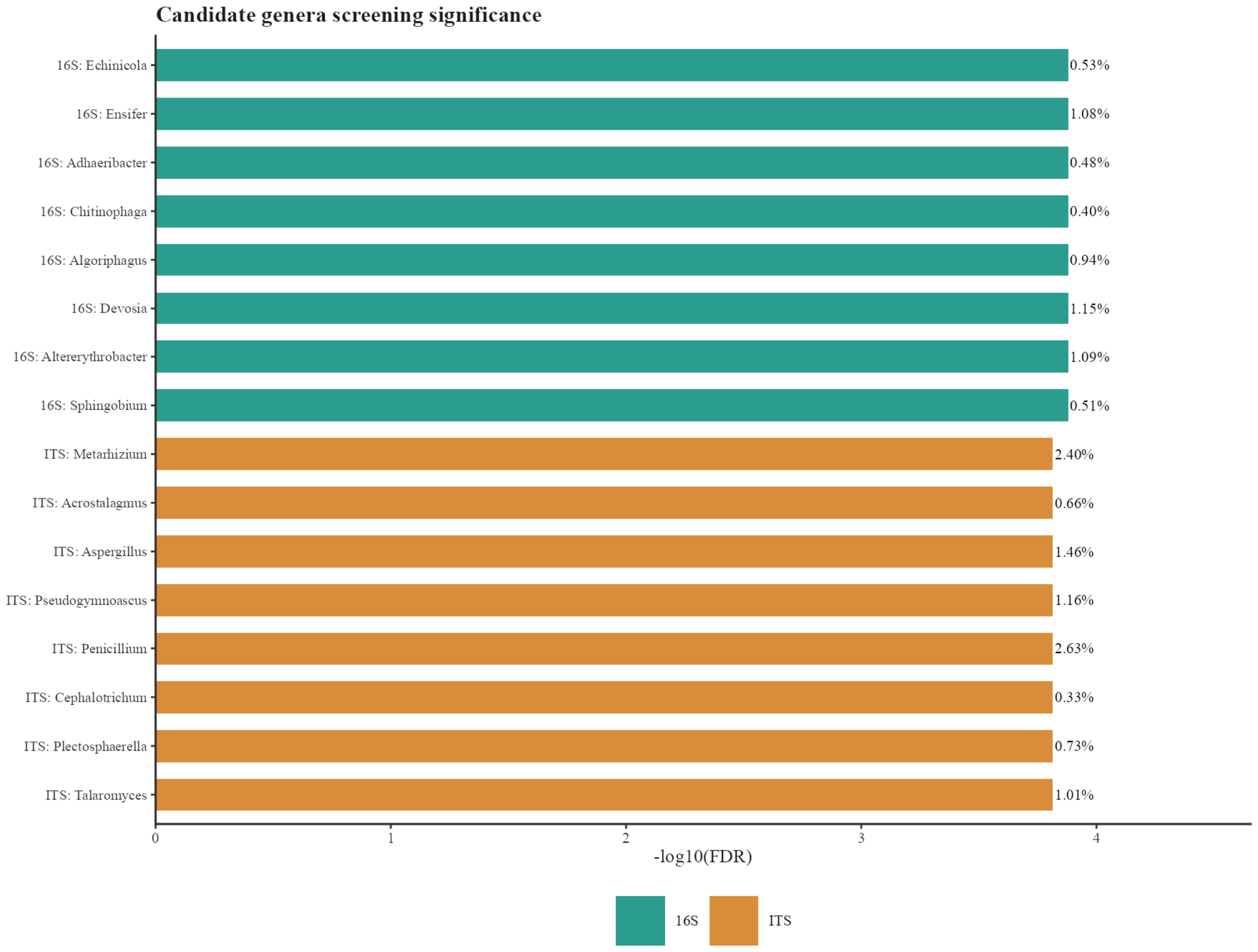

Figure S4. Screening-level candidate bacterial and fungal genera. Bars show $-\log_{10}$(FDR-adjusted Kruskal–Wallis screening value), and labels give group-mean relative abundance. Teal and orange identify 16S and ITS candidates. No confirmatory differential-abundance model was available; candidates are hypothesis-generating only.

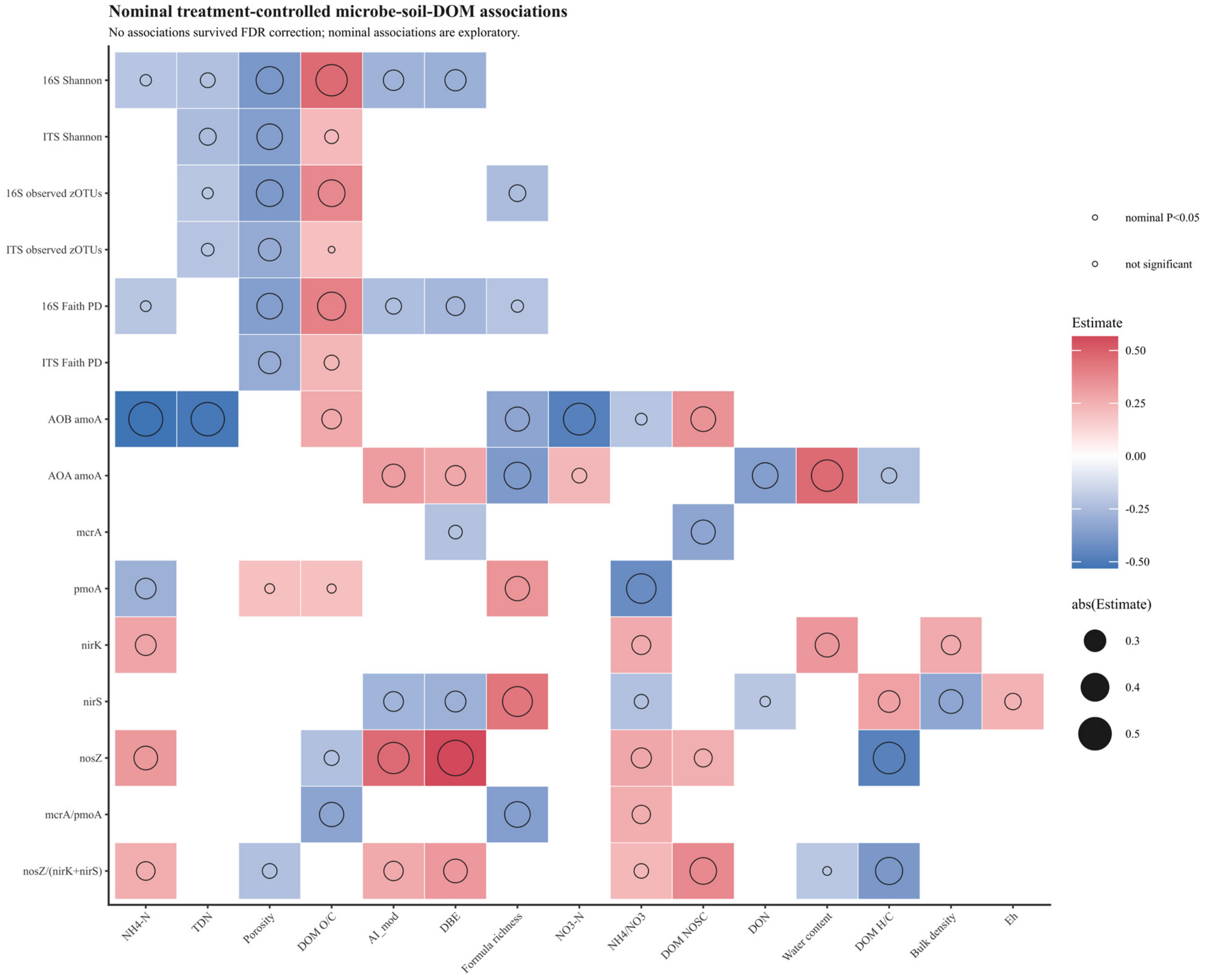

Figure S5. Nominal treatment-controlled associations among microbiome, soil, qPCR and DOM variables. Associations were calculated from treatment-group residuals. Cell colour shows direction and magnitude; symbol size shows absolute magnitude. Microbiome richness uses observed zOTUs. No residual association remained significant after FDR correction.

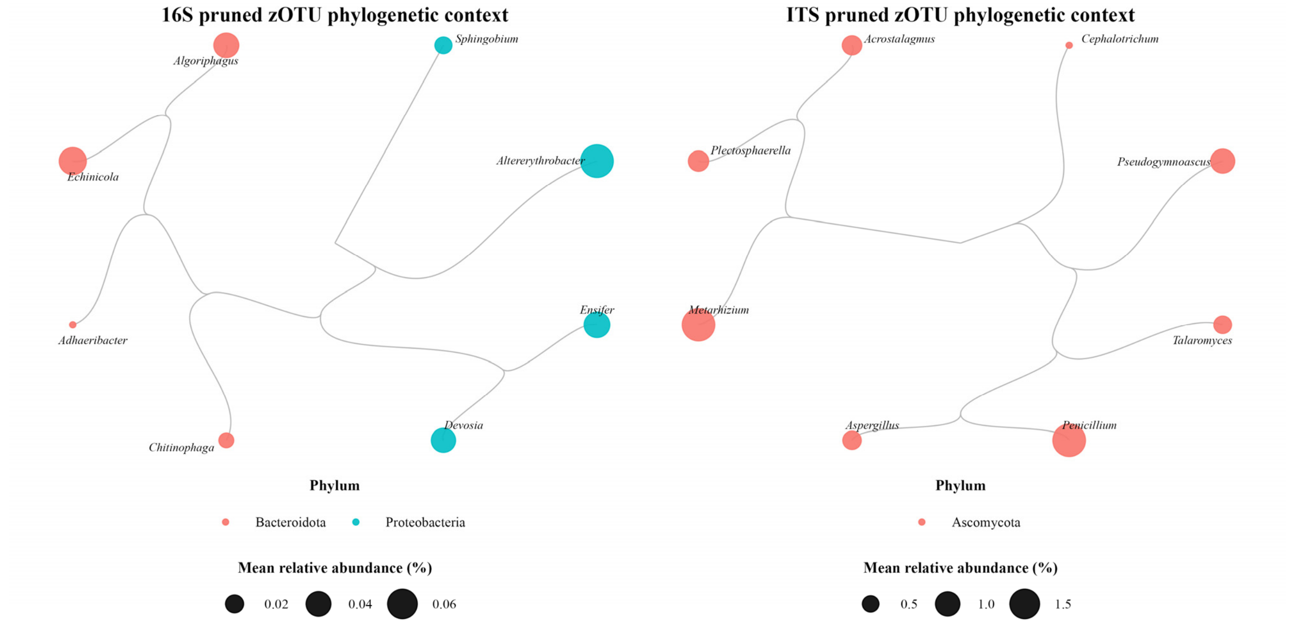

Figure S6. Pruned phylogenetic context for selected bacterial and fungal zOTUs. Representative 16S and ITS tips are labelled by the available taxonomic assignment, coloured by phylum and scaled by mean relative abundance. The trees provide taxonomic context only and are not a comprehensive phylogeny.

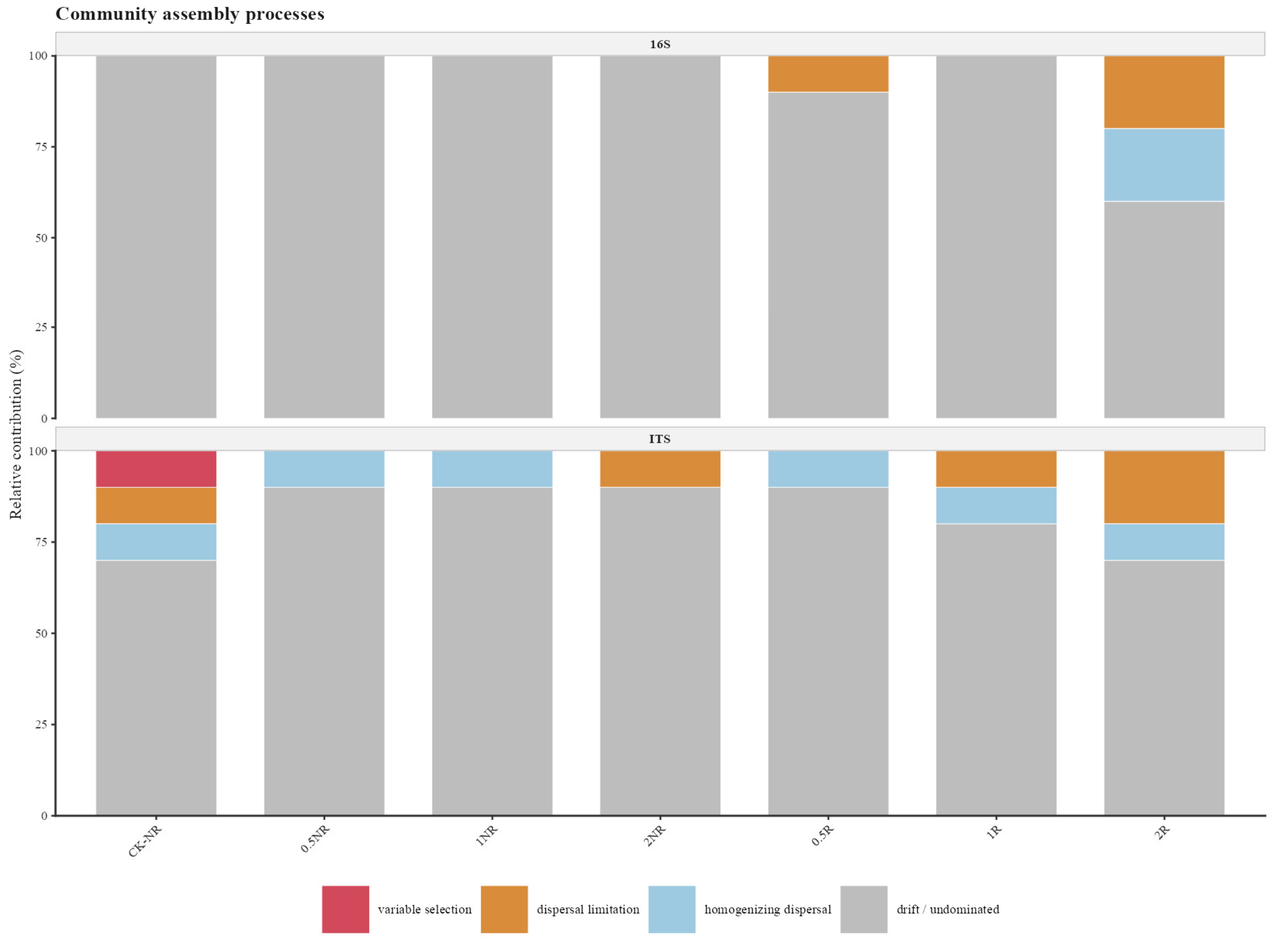

Figure S7. Exploratory community-assembly summaries for 16S and ITS datasets across observed groups. Bars show process categories assigned by the retained null-model workflow. The outputs are descriptive and do not by themselves demonstrate ecological mechanism.

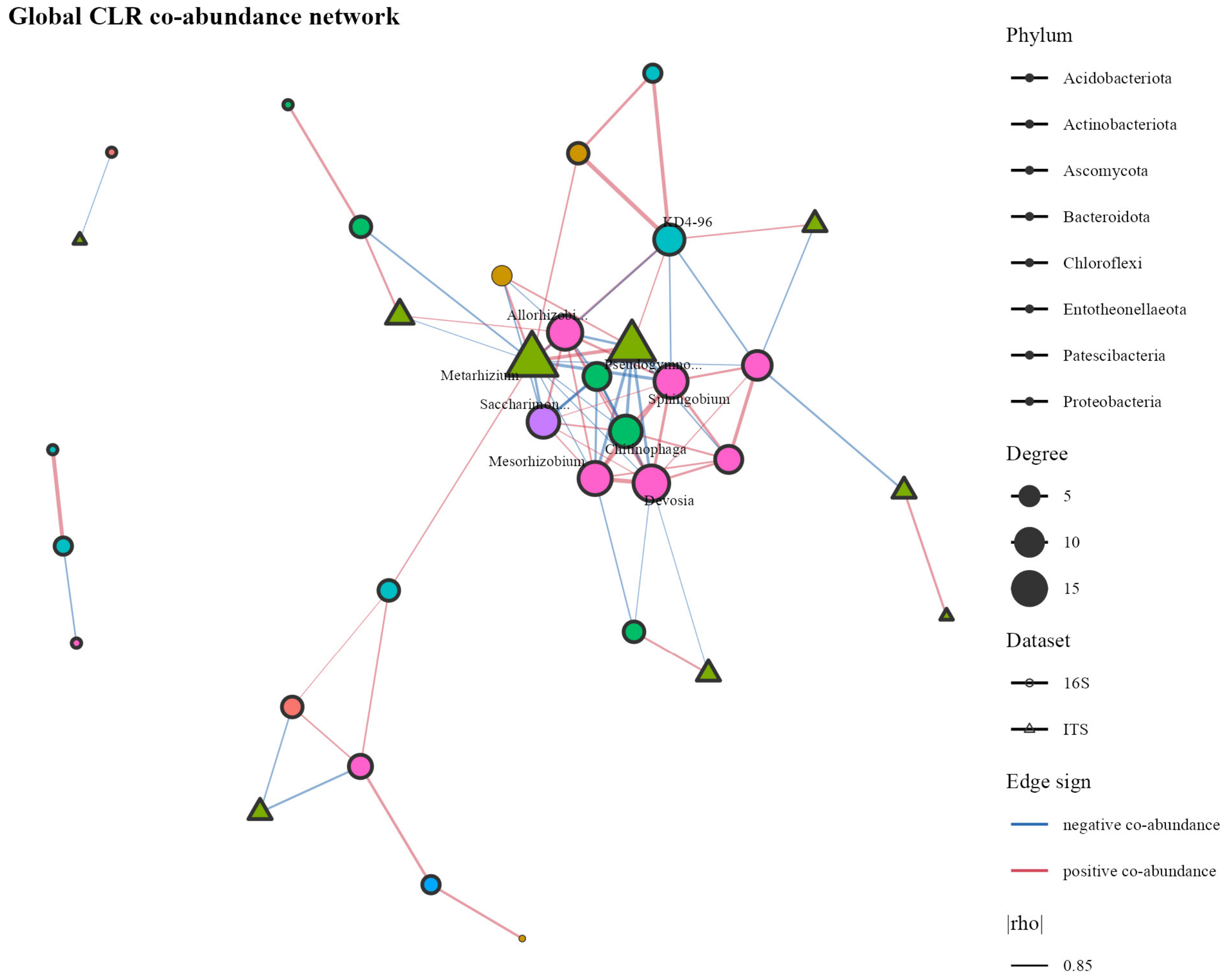

Figure S8. Global centred-log-ratio co-abundance network. Circles and triangles represent 16S and ITS taxa, node colour denotes phylum, node size denotes degree, and red/blue edges denote positive/negative co-abundance. Edges are associations, not direct ecological interactions or causality.

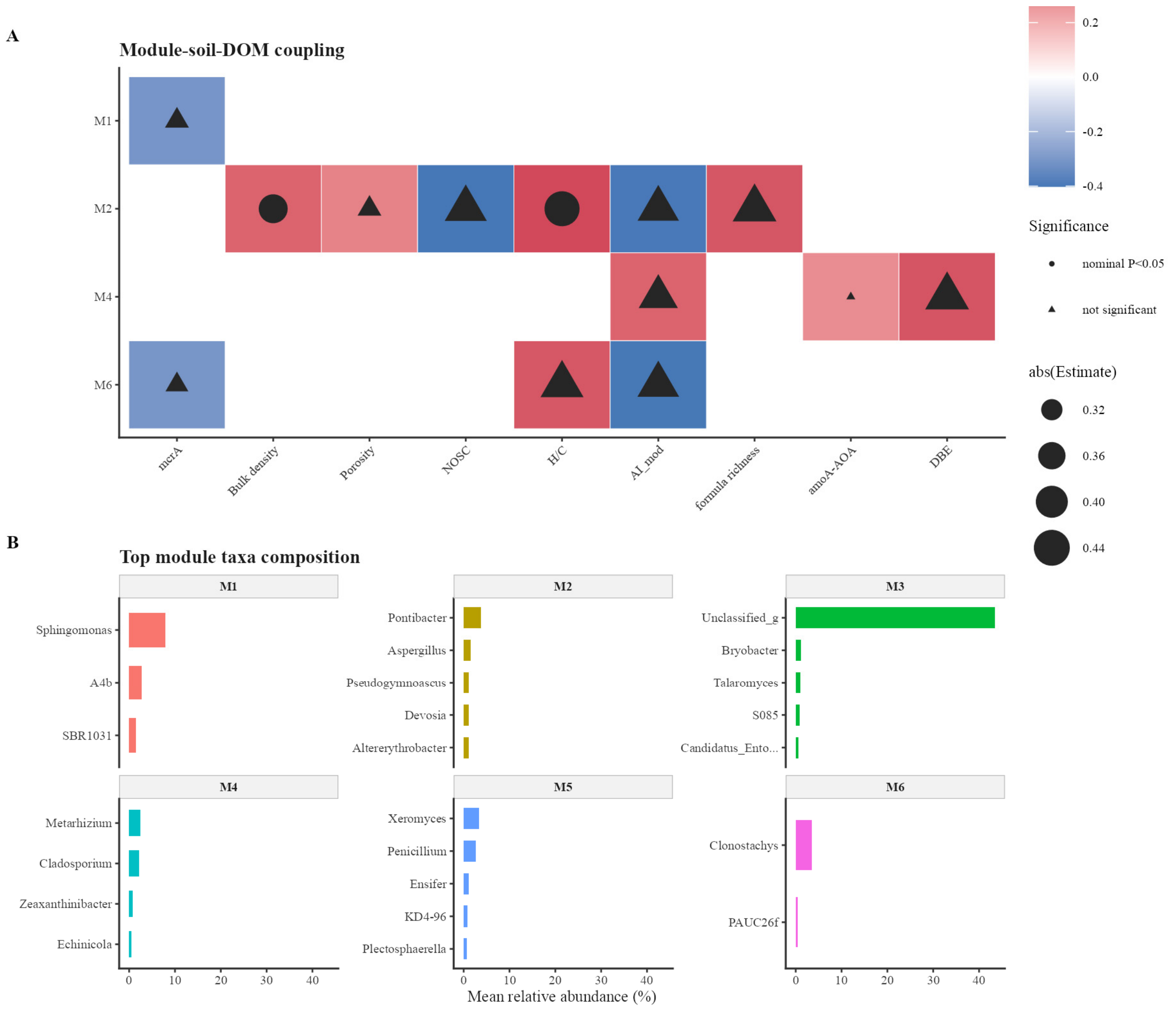

Figure S9. Exploratory coupling of microbiome modules with soil and DOM variables. (A) Module-level nominal associations. (B) Leading taxa by module. No cross-layer residual association survived FDR correction; the panel is hypothesis-generating.

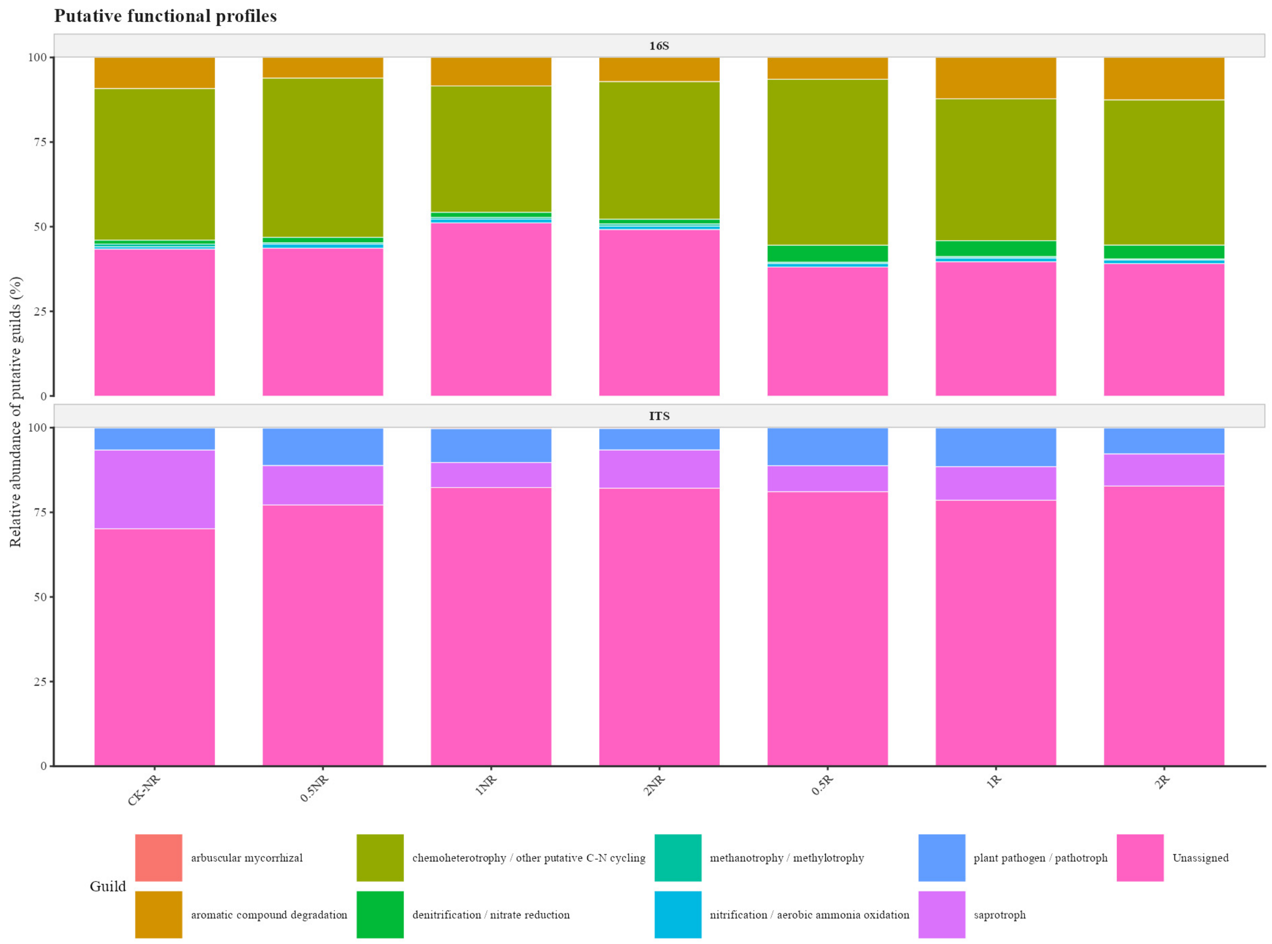

Figure S10. Putative taxonomic functional profiles for bacterial and fungal communities. Categories were assigned from taxonomy and are not metagenomic measurements of genes, transcripts or rates.

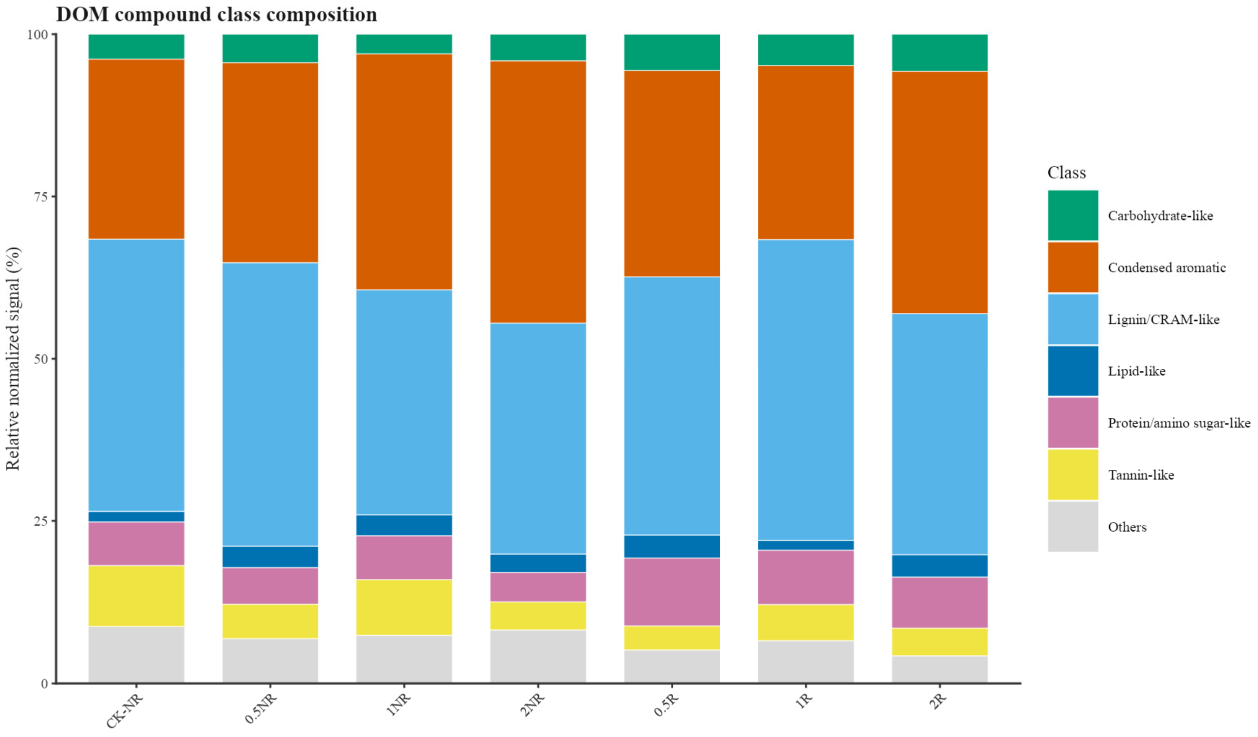

Figure S11. D90 DOM assigned-formula class composition across the seven observed groups. Bars reproduce relative normalized-signal summaries. These profiles describe the detected ionizable DOM fraction and are not concentration-calibrated.
